# A novel host-based strategy for detecting low-grade prosthetic joint infection using immune cell pattern in synovial fluid

**DOI:** 10.1186/s10020-026-01461-0

**Published:** 2026-03-28

**Authors:** Marketa Trajerova, Eva Kriegova, Bishu Shrestha, Milos Kudelka, Jakub Savara, Jiri Gallo

**Affiliations:** 1 Department of Immunology, Faculty of Medicine and Dentistry, Palacký University Olomouc and University Hospital Olomouc, Hnevotinska 3, 779 00 Olomouc, Czech Republic; 2 Department of Computer Science, Faculty of Electrical Engineering and Computer Science, VSB-Technical University of Ostrava, Ostrava, Czech Republic; 3 Department of Orthopaedics, Faculty of Medicine and Dentistry, Palacký University Olomouc and University Hospital Olomouc, 779 00, Zdravotniku 248/7, Olomouc, Czech Republic

**Keywords:** Multiparametric biomarker detection, Cellular biomarkers, Arthroplasty, Neutrophil immunophenotypes, Implant wear particles

## Abstract

**Background:**

Reliable biomarkers for the routine diagnostics of low-grade prosthetic joint infection (PJI) remain an unmet clinical need.

**Methods:**

The cellular and soluble content of synovial fluid (SF) from 108 patients was investigated using flow cytometry and ELISA. The study cohort included 44 patients with total knee/hip arthroplasty (16 with PJI, 8 with low-grade PJI with low α-defensin/CRP levels, 20 with osteolysis/aseptic loosening) and 64 controls with osteoarthritis with/without infection (OA-INF/OA: 28/36). To investigate the interconnectivity between wear particle-associated mechanisms and infection-associated patterns, primary human OA fibroblast-like synoviocytes and OA chondrocytes cultured with/without Ti_6_Al_4_V alloy were analysed through RNAseq. A multiparametric patient similarity network (PSN) approach was used to evaluate the results.

**Results:**

A multiparametric PSN revealed that the combination of a low proportion of dendritic cells (cDC2s), HLA-DR^+^ natural killer (NK) cells, a high proportion of NK cells from lymphocytes, a higher percentage of CD88^+^ cDC1 cells, and neutrophil (NEU) immunophenotypes has demonstrated outstanding performance in detecting PJI, including low-grade PJI, comparing to sterile inflammation induced by wear particles. Moreover, cellular biomarkers on NEUs differed in PJI with culture positivity, low-grade PJI and aseptic loosening. In addition, the gene expression of *IL6*, *CXCL1/3/5/6/8*, *CCL2/7,* and *MMP9* of synoviocytes and chondrocytes stimulated by Ti_6_Al_4_V alloy particles confirms the interconnectivity between infection- and wear particle-associated mechanisms, which discriminates some traditional soluble infection-associated biomarkers.

**Conclusion:**

We propose a novel host-based strategy for detecting low-grade PJI based on composition and immunophenotype of immune cells derived from SF, shifting the emphasis from pathogen detection and ambiguous clinical manifestations to cellular biomarkers. Our findings showed that cellular biomarkers may be beneficial for the detection of PJI, including low-grade PJI, even in the presence of wear particles activating inflammatory responses mimicking infectious conditions. This host-based approach may improve diagnostic strategies for low-grade PJI.

**Supplementary Information:**

The online version contains supplementary material available at 10.1186/s10020-026-01461-0.

## Introduction

Prosthetic joint infection (PJI) is a rare, yet devastating complication following total joint arthroplasty (TJA). It has an incidence of 1–2%, and is associated with substantial patient morbidity, and mortality (Fischbacher and Borens 2019). Despite extensive efforts, the diagnosis of PJI–—especially its low-grade forms–—remains a tremendous challenge.

Low-grade PJI is increasingly recognized as a distinct pathophysiological condition rather than simply a less severe form of PJI. It is characterised by a subtle clinical course, minimal inflammatory response, and often inconclusive laboratory findings (Ince et al. 2004). Conventional diagnostic approaches that rely on direct detection of pathogens by culture or nucleic acid amplification may fail in low-grade PJI due to the low bacterial burden and virulence, the presence of biofilm-forming microorganisms, variability in host immune status or prior antibiotic exposure (Elsissy et al. 2020; Medzhitov and Iwasaki 2024; Wu et al. 2025). In addition, the diagnostics of PJI is complicated by a chronic sterile inflammatory reaction to prosthesis wear particles, which can mimic infectious conditions, blurring the lines between septic and aseptic failure (Hodges et al. 2021; Matzinger 2002). Despite novel biomarkers of PJI, such as small antimicrobial peptides (e.g. α-defensins), the long pentraxin PTX3 (Loppini et al. 2023) and calprotectin (Belkum et al. 2022), the diagnostics of low-grade PJI is challenging. Reasons include limited validation in low-grade PJI cohorts, the lack of harmonized cut-offs across assays, and reduced specificity in the setting of aseptic wear particle-induced inflammation, where overlapping innate immune pathways can yield false-positive results (e.g., α-defensin false-positivity in metallosis or pronounced polyethylene wear) (Heckmann et al. 2023).

In this study, we adopted a host-response strategy for detecting low-grade PJI. The conceptual premise is that synovial immune-cell composition and activation phenotypes integrate the net effect of pathogen presence, biofilm activity, host immune status, and prior antibiotic exposure, i.e. factors that can each compromise direct pathogen detection. This approach is particularly relevant to low-grade PJI, where bacterial burden is low and inflammation is often subtle, making cultures, nucleic-acid assays, and single soluble biomarkers prone to false-negative or inconclusive results. We, therefore, profiled synovial immune-cell immunophenotypes, with emphasis on neutrophils (NEUs) and the monocyte–macrophage (MON–Mϕ) lineage cells as key first-line innate effector cells at the implant–tissue interface. In addition, we applied unsupervised multiparametric patient similarity network (PSN) to capture host related immune patterns that may improve discrimination across overlapping clinical phenotypes compared with univariate thresholds. Finally, to contextualize potential distortion caused by aseptic particle-induced inflammation, we examined wear-associated inflammatory programming in primary fibroblast-like cells (FLSs) and chondrocytes following stimulation with Ti_6_Al_4_V alloy particles.

## Material and methods

### Patients and sample pre-processing

The study cohort consisted of 108 patients (men/women: 66/42, median age: 66 years) who were recruited between November 2020 and November 2024. Of the patients with total knee/hip arthroplasty due to the end-stage OA, 24 were diagnosed with PJI (group PJI-all) and 20 were control patients with osteolysis/aseptic loosening (group OL/AL). The distinction between septic and aseptic cases was made according to contemporary definitions of PJI and non–implant-related infections (McNally et al. 2021; Mathews et al. 2010). In this line, patients with PJI were stratified into two subgroups as follows: i) patients with definite PJI (*n* = 16), defined as positive microbial cultivation from SF and/or periprosthetic tissue and/or positive histology consistent with infection and ii) patients classified according to the 2021 EBJIS criteria (McNally et al. 2021) as ‘infection confirmed’ or ‘infection likely’ (n = 8) and presenting without acute clinical signs (systemic and/or local) were included. In addition, two patients categorized as ‘infection unlikely’ but exhibiting acute clinical signs were also included. These cases typically showed a low-grade inflammatory profile with elevated yet often subthreshold synovial white blood cell counts, intermediate NEU proportions (commonly 40–65%), borderline or inconclusive α-defensin and/or C-reactive protein (CRP) results, and frequently negative cultures. Two control groups were included: OA with noninfectious joint effusion (OA; n = 36) and patients with OA and concomitant joint infection unrelated to implants (OA-INF; n = 28) (Mathews et al. 2010). Samples were obtained pre-operatively or intraoperatively before joint capsule incision. For more details on patient characteristics and detected microbes, see Table 1 and Supplementary Table S1.

**Table 1:** Detailed characteristics of enrolled patients with prosthetic infection (PJI-all), its subgroups with microbiologically confirmed prosthetic infection (PJI) and low-grade prosthetic joint infections (LG-PJI), patients with osteolysis and/or aseptic loosening (OL/AL) and control groups of patients with osteoarthritis with infection (OA-INF) and osteoarthritis (OA). The full table of measured parameters can be found in Supplementary Table S3

	**PJI-all**	**PJI-all**		**OL/AL**	**OA-INF**	**OA**
		**LG-PJI**	**PJI**			
No. of patients	24	8	16	20	28	36
Sex (men/women)	17/7	7/1	10/6	12/8	13/15	24/12
Knee/hip	20/4	7/1	13/3	13/7	28/0	36/0
Age at SF sampling (years): mean ± 95% CI; [min–max]; *NA*	70.9 ± 3.5; [50.0–81.0]; *0*	70.9 ± 4.8; [63.0–78.0]; *0*	70.9 ± 5.1; [50.0–81.0]; *0*	70.2 ± 5.8; [44.0–90.0]; *0*	61.0 ± 5.1; [35.0–85.0]; *0*	60.8 ± 3.9; [43.0–84.0]; *0*
Time since joint difficulties in months: mean ± 95% CI; [min–max]; *NA*	16.5 ± 9.5; [1.0–72.0]; *4*	36.0 ± 26.4; [12.0–72.0]; *2*	8.1 ± 5.9; [1.0–36.0]; *2*	16.2 ± 8.7; [0.0–60.0]; *2*	4.1 ± 1.8; [0.0–12.0]; *3*	19.6 ± 10.7; [1.0–120.0]; *2*
Time since implant placement in months: mean ± 95% CI; [min–max]; *NA*	65.4 ± 44.5; [1.0–336.0]; *1*	38.7 ± 72.6; [1.0–216.0];* 1*	77.1 ± 59.7; [5.0–336.0]; *0*	23.6 ± 28.2; [1.0–216.0]; *2*	–	–
Cement YES/NO/HYBRID/*NA*	21/2/0/*1*	7/0/0/*1*	14/2/0/*0*	4/16/0/*0*	–	–
Cement type: Biomet Optipac/Copal G + C/Refobacin/none/*NA*	6/1/3/2/*12*	3/1/0/0/*4*	3/0/3/2/*8*	3/0/1/5/*11*	–	–
BMI (kg/m^2^): mean ± 95% CI; [min–max]; *NA*	31.0 ± 2.3; [22.0–45.9]; *0*	30.5 ± 4.3; [24.0–39.5]; *0*	31.3 ± 3.1; [22.0–45.9]; *0*	30.2 ± 2.0; [24.1–40.9]; *2*	30.3 ± 3.1; [19.5–39.5]; *13*	29.2 ± 2.1; [18.4–50.3]; *4*
KL grade of OA^a^: 1/2/3/4	–	–	–	–	0/16/12/0	0/20/16/0
Range of joint damage^b^: 0/1/2/3	–	–	–	–	0/6/20/2	0/8/25/3
Pain level in VAS^c^: mean ± 95% CI; [min–max]; *NA*	4.5 ± 0.5; [3.0–7.0]; *0*	5.0 ± 1.1; [3.0–7.0]; *0*	4.2 ± 0.6; [3.0–6.0]; *0*	4.3 ± 0.8; [0.0–8.0]; *0*	3.8 ± 0.5; [2.0–6.0]; *1*	3.1 ± 0.5; [1.0–7.0]; *7*
Pain level^d^: 0/1/2/3/*NA*	0/0/19/5/*0*	0/0/7/1/*0*	0/0/12/4/*0*	1/2/13/4/*0*	0/8/18/0/*2*	0/13/18/1/*4*
Fluid volume (ml): mean ± 95% CI; [min–max]; *NA*	21.7 ± 8.6; [2.0–65.0]; *0*	31.8 ± 21.4; [2.5–65.0]; *0*	16.7 ± 8.5; [2.0–55.0]; *0*	17.5 ± 6.5; [1.2–50.0]; *0*	49.3 ± 18.4; [10.0–250.0]; *0*	31.0 ± 7.0; [4.0–100.0]; *0*
Viscosity: low/normal/high	13/9/2	7/1/0	6/8/2	11/8/1	17/10/1	18/17/1
Colour: transparent/yellow/cloudy/orange/*NA*	0/1/16/7/*0*	0/0/2/6/*0*	0/1/5/10/*0*	0/0/12/8/*0*	3/1/24/0/*0*	22/10/2/2/*0*
Absolute CD45^+^ cell count in SF × 10^9^/l; mean ± 95% CI; [min–max]; *NA*	25.6 ± 17.5; [0.1–149.1]; *0*	2.4 ± 2.0; [0.5–6.3]; *0*	37.3 ± 24.9; [0.1–149.1]; *0*	1.2 ± 1.0; [0.1–9.7]; *0*	17.3 ± 6.4; [0.2–59.3]; *0*	0.5 ± 0.2; [0.1–2.9]; *0*
LYM [%]: mean ± 95% CI; [min–max]; *NA*	14.1 ± 8.3; [0.5–67.4]; *0*	8.3 ± 3.9; [3.1–15.3]; *0*	17.0 ± 12.6; [0.5–67.4]; *0*	46.5 ± 11.1; [6.4–89.1]; *0*	7.6 ± 3.2; [0.2–29.5]; *0*	34.8 ± 6.5; [4.5–84.1]; *0*
MON–Mϕ [%]: mean ± 95% CI; [min–max]; *NA*	11.9 ± 4.2; [0.5–28.8]; *0*	12.5 ± 8.7; [1.0–28.8]; *0*	11.5 ± 5.3; [0.5–27.2]; *0*	32.9 ± 10.6; [6.0–77.9]; *0*	13.7 ± 5.0; [0.9–66.8]; *0*	50.5 ± 6.8; [12.3–92.2]; *0*
NEU [%]: mean ± 95% CI; [min–max]; *NA*	73.2 ± 11.3; [4.2–98.1]; *0*	78.2 ± 10.5; [59.4–92.2]; *0*	70.7 ± 16.8; [4.2–98.1]; *0*	19.5 ± 6.6; [2.5–47.0]; *0*	70.9 ± 7.1; [1.7–97.8]; *0*	13.6 ± 3.6; [1.4–44.8]; *0*

^a^KL grade of OA was classified according to the Kellgren–Lawrence Grading Scale

^b^Severity of OA was estimated from radiography and categorised as 0 = early-stage OA; 1 = either medial or lateral tibiofemoral joint clearly affected by KOA grade 2 or higher; 2 = both compartments (medial/lateral tibiofemoral/patellofemoral) affected by KL grade 2 or higher; 3 = all three compartments were affected by KL grade 2 or higher

^c^Categorization of pain level based on the self-reported 11–point visual analogue scale (VAS): 0 = no pain (VAS 0–1); 1 = mild pain (VAS 2–3); 2 = moderate pain (VAS 4–5); 3 = severe pain (VAS ≥ 6)

^d^Pain level according to self-reported 11–point visual analogue scale (VAS)

*LYM=* lymphocytes, *MON–Mϕ=* monocyte–macrophage lineage, *NEU=* neutrophils, *NA=* not available

### Processing of samples and analysis of synovial fluid-derived immune cells through flow cytometry

The SF samples were carefully examined (volume/colour/opacity/viscosity) and processed within 4 h after aspiration. Total immune cell counts were calculated using CountBright absolute counting beads (Thermo Fisher, USA) from the native sample and expressed as the number of cells per unit of volume (cells/l). After centrifugation, the cell pellet was used for flow cytometry analysis using FACSAria fusion (BD Biosciences, USA)(Mikulkova et al. 2024; Trajerova et al. 2022; Kriegova et al. 2018). For used antibodies and the gating strategy, see Supplementary Table S2, Supplementary Figure S1 and Figure S2. The main immune cell populations (lymphocytes (LYM), MON–Mϕ lineage cells, NEUs) were calculated as a percentage of CD45^+^ cell singlets. The percentages of subpopulations were calculated either as a part of parental populations or as a percentage of all CD45^+^ cell singlets. In all experiments, a minimum of 10,000 cells from each of the main populations was measured, and a cut-off of 500 events was used to evaluate the activation markers. Activation status was determined either by the percentage of cells expressing the selected marker (%) or as the median fluorescence intensity (MFI). The cell supernatants were stored at − 80 °C in aliquots until analysis.

### Cell line cultivation in the presence of Ti_6_Al_4_V alloy particles

Primary human cell lines of OA FLSs (408OA-05A, Sigma-Aldrich, USA) and OA chondrocytes (402OA-05A, Sigma-Aldrich) were cultivated in Dulbecco’s Modified Eagle Medium/F12, supplemented with 10% heat-inactivated foetal bovine serum (both Thermo Fisher Scientific, USA) and 100 U/ml of penicillin and 100 μg/ml streptomycin (Sigma-Aldrich) 24 h with and without Ti_6_Al_4_V alloy particles (grade 23/15–45 μm, uncoated, Tekna Advanced Materials INC., Canada) at 37 °C and 5% CO_2_. To remove endotoxin, the particles were boiled in 1% acetic acid for 90 min, washed three times with distilled H_2_O and autoclaved at 135 °C for 3 h as previously reported (Choi et al. 2005). The presence of endotoxin was analysed using the Pierce™ Chromogenic Endotoxin Quant Kit (Thermo Fisher Scientific). The particles were then washed several times in phosphate-buffered saline and resuspended by pipetting at the desired concentration immediately before culturing. The cells were counted using a fluorescent cell counter (Cellometer K2, Nexcelom Biosciences, USA) and seeded for FLS cultivation at a density of 9,000 cells per cm^2^ and for chondrocyte cultivation at a density of 15,000 cells per cm^2^ in 6-well polystyrene plates (CELLSTAR multiwell culture plates, Greiner bio-one, Austria). The Ti_6_Al_4_V alloy particles were added to the cell lines at a concentration of 0.65 mg/cm^2^ and cultivated for 24 h at 37 °C in a humidity-controlled incubator with 5% of CO_2_ supply in triplicates. The cultivations of FLSs and chondrocytes without Ti_6_Al_4_V alloy particles were used as negative controls. Cell viability was assessed using the RealTime-Glo MT Cell Viability Assay (Promega, USA) and measured with Cytation 5 (Agilent, USA). Under the applied conditions, the difference in viability between Ti-treated and untreated cells was < 10%. Cells were inspected for adhesion and morphology before and after culturing with/without Ti_6_Al_4_V alloy particles and used for isolation of total RNA.

### Whole transcriptome analysis through bulk RNAseq of cultured cells

Total RNA was isolated from the cultured FLS/chondrocytes using the RNAqueous-Micro Total RNA Isolation Kit (Thermo Fisher Scientific) (Mikulkova et al. 2024). The libraries were prepared using the NEBNext® poly(A) mRNA Library Prep Kit (New England Biolabs, USA) and sequenced on a NovaSeq 6000 (Illumina Inc., San Diego, CA, USA) using paired-end sequencing (2 × 151 bp) at 30 × coverage. Reads were demultiplexed (bcl2fastq), FASTQ files were aligned to the hg38 human genome and mapped to genes (STAR tool, https://github.com/alexdobin/STAR) using Ensembl gene annotation (www.ensembl.org/info/genome/genebuild/index.html). The ThreeDRNAseq (3DRNAseq) R package was used for the quantification of the RNAseq reads.

### Protein-level measurement in synovial fluid

Levels of procalcitonin (PCT; BioTechne, USA), CRP (BioTechne), presepsin (sCD14; BioTechne), α-defensins (HNP1-3; Hycult Biotech, Netherlands), PTX3 (Hycult Biotech), soluble TREM1 (sTREM1; Hycult Biotech) and CXCL13 (BioTechne) were measured using ELISA according to the manufacturer’s recommendations.

### Patient similarity network construction

In this study, we analysed flow cytometry data (i.e. the cell distribution or expression of markers on cells) using a PSN as a framework for unsupervised learning. This network captures similarities between pairs of patients and was constructed from the original vector data representing individual patients (parameter sets). We used the local representative network (LRNet) method (https://homel.vsb.cz/~kud007/lrnet_files/) (Ochodkova et al. 2017) to construct the network, which is based on the analysis of similarities between nearest neighbours. A key advantage of networks, including PSNs, is that they can be easily visualised, even with a relatively small number of vertices (in the thousands), and even when the input patient data has a high dimension (i.e. tens to hundreds of parameters). Patient data visualised in this way can then be interpreted relatively easily, particularly when studying groups of similar patients.

The PSN (mathematical graph) is a structure containing vertices representing patients and links between pairs of patients, where the strength of the link expresses the similarity between the pair. The first step in constructing the network is to calculate these similarities. Our approach uses a Gaussian function to measure similarity by transforming the Euclidean distance into a similarity between patients represented as vectors of their individual parameters, rescaled to the interval [0,1] (Ochodkova et al. 2017). The second step is to select pairs of patients who are linked in the network. These pairs are determined by similarity; the LRNet algorithm computes each patient's representativeness and, based on this, calculates the number of nearest neighbours with whom they are linked in the network. The next step after constructing the PSN is to detect groups of similar patients (with similar parameter vectors). Methods for detecting so-called communities are used for networks, in our case referred to as patient clusters. The Louvain method we use, based on modularity optimisation (a measure of the quality of the network's community structure), detects clusters of different sizes, with each patient belonging to only one cluster. Its main advantage is its speed and comprehensibility of output. To identify a final network with desirable, clinically useful properties, we constructed networks and detected clusters across various parameter combinations. We then measured the following properties of the networks and clusters obtained in this way: modularity (structural quality of clustering), silhouette (unambiguity of patient assignment to clusters), reliability of centroids (average patients in clusters), and variance of values in clusters (standard deviation of individual parameters in a cluster). There was no missing data in the input to the final network in this study, and it was the most balanced of all the networks we tested with respect to the above measurements.

### Data analyses

The Shapiro–Wilk normality test, Mann–Whitney U test, Kruskal–Wallis test with Bonferroni correction, receiver operation curves (ROC) for individual markers and combined ROC analysis with a multivariable RIDGE logistic regression model for multiple markers, where the area under the curve (AUC) was estimated by nonparametric DeLong’s method and cut-off values for markers determined by Youden’s J statistic, were performed using Python (version 3.10, https://www.python.org/) and R software (version 4.2.3, http://www.r-project.org/). P-values of ≤ 0.050 were considered significant.

## Results

### Patients

Of the 24 patients with PJI (PJI-all), 16 had positive microbial cultivation and 8 had low-grade PJI with negative microbial cultivation and low α-defensin/CRP levels. When evaluating only according to the SF-related criteria of PJI, 11 patients with a positive culture (PJI) had ‘infection confirmed’ status and 5 ‘infection unlikely’ status (McNally et al. 2021). Of the eight patients with low-grade PJI with negative microbial cultivation, six had ‘infection confirmed’ status and two ‘infection unlikely’ status. For levels of α-defensins, leukocytes, NEUs and CRP in SF in individual patients, see Supplementary Table S1.

### Detection of cellular and soluble biomarkers associated with prosthetic joint infection

**Fig. 1:**
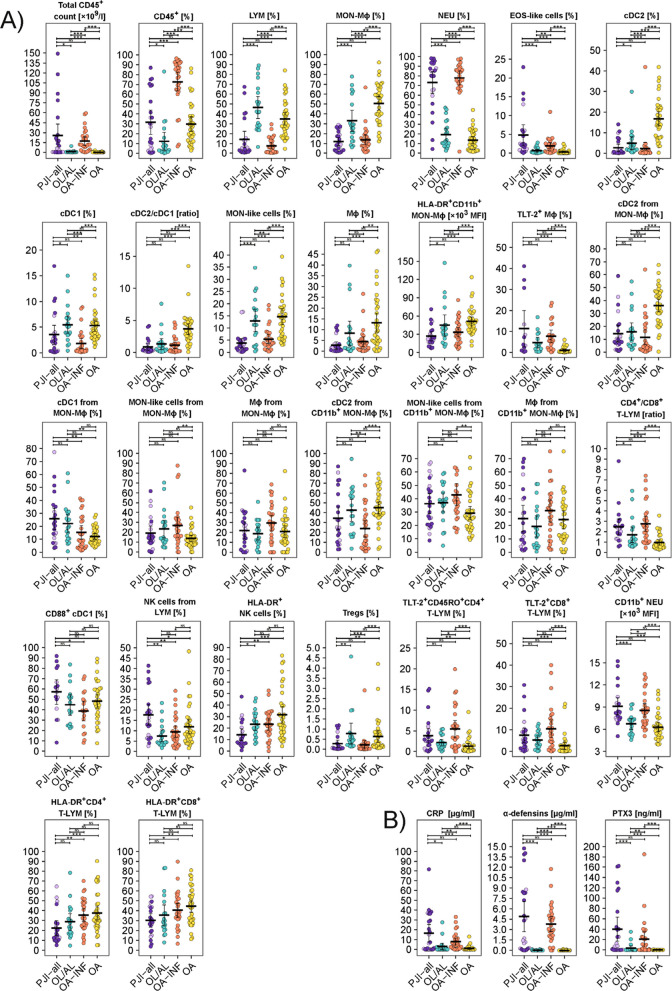
Distribution (**A**) of counts and immunophenotypes of synovial fluid (SF)-derived immune cells and (**B**) soluble markers in SF in groups of patients with and without infection. The full table of measured parameters can be found in Supplementary Table S3. Horizontal bar represents mean, and whiskers represent 95% CI. P-values are represented as follows: *** *p* ≤ 0.001; ** 0.001 < *p* ≤ 0.01 and * 0.01 < *p* ≤ 0.05. Legend: PJI-all = patients with microbiologically confirmed prosthetic joint infection (PJI; dark violet) and with low-grade PJI (light violet); OL/AL = patients with osteolysis and/or aseptic loosening of the implant; OA-INF = patients with osteoarthritis and joint infection; OA = patients with osteoarthritis; NS = no significance

In infection groups (PJI-all and OA-INF), higher total counts and the percentage of CD45^+^ immune cells, NEUs and eosinophil (EOS)-like subpopulations and lower percentages of MON–Mϕ and LYM were detected comparing to non-infection groups (Fig. 1, Supplementary Table S3). When analysing the MON–Mϕ lineage cells, lower percentages of CD11b^+^ myeloid dendritic cells (DCs; type cDC2) and higher percentage of CD11b^−^ DCs (cDC1) were detected in the PJI-all and OA-INF groups compared with the non-infection groups (Fig. 1). For distribution of other immune cells in studied groups, see Fig. 1 and Fig. 3. A subanalysis of the studied markers did not reveal any relationship with gender, age, or location of affected joint.

Regarding soluble mediators, SF concentrations of CRP, PTX3 and α-defensins were higher in the PJI-all and OA-INF groups than in the non-infection groups (Fig. 1, Supplementary Figure S3). Using ELISA with a limit of detection (LOD) of 156 pg/ml, 39/44 PJI-all samples were positive for α-defensins. Using a cut-off for lateral flow tests (LOD 1,560 ng/ml) (Bonanzinga et al. 2019), only 13/44 PJI-all samples were positive, of which 10/16 samples were from the PJI group and 3/8 samples from the low-grade PJI group (Supplementary Table S1).

### Patient similarity network analysis on cellular and soluble markers of prosthetic joint infection

**Fig. 2:**
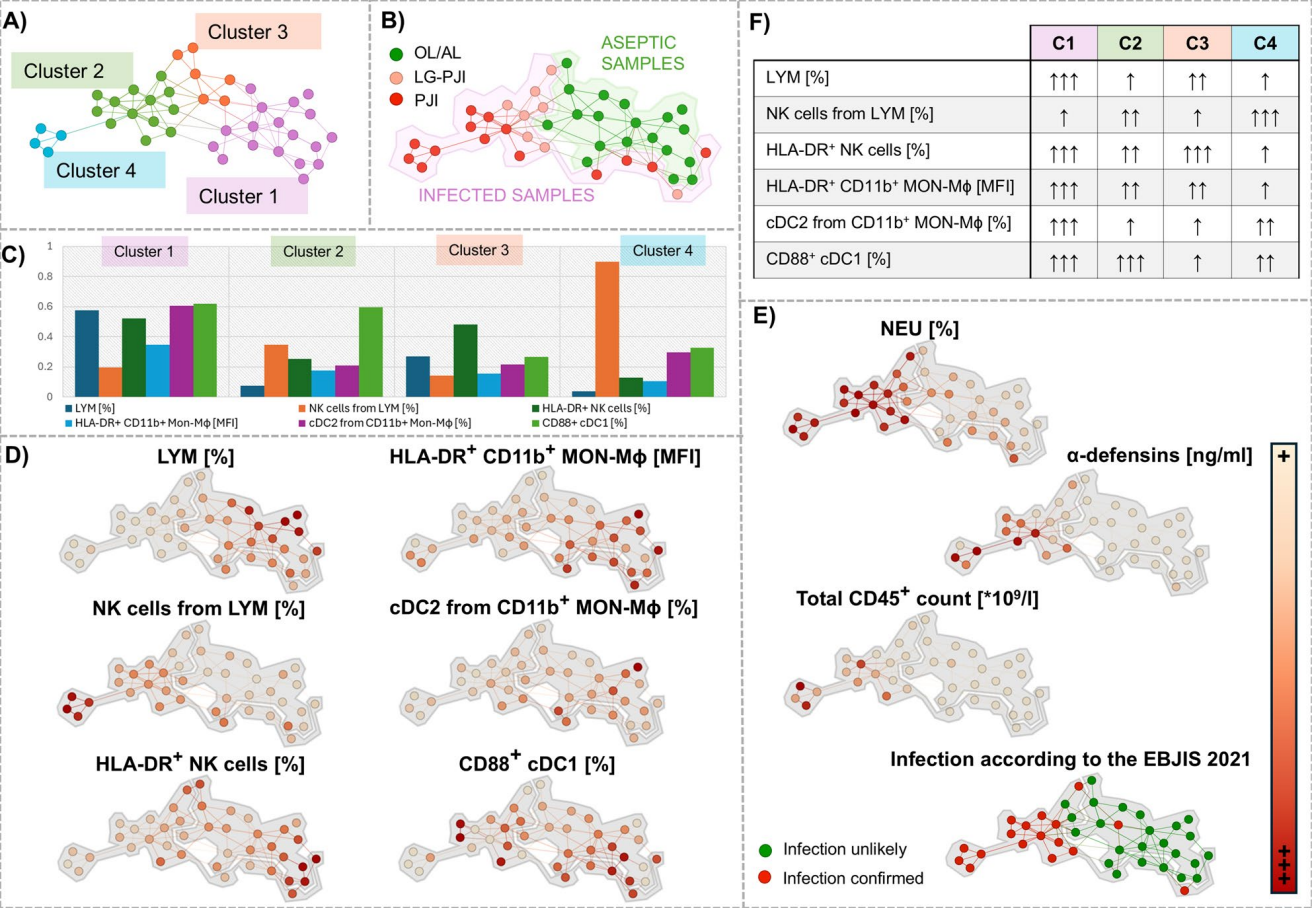
Characteristics of the patient similarity network constructed for patients with total joint arthroplasty for the visualisation of prosthetic joint infection (PJI; confirmed PJI and low-grade LG-PJI) and aseptic conditions (osteolysis/aseptic loosening; OL/AL). (**A)** Four clusters based on the similarity of used parameters were obtained. (**B)** Distribution of the patient subgroups in the network; the area of patients with aseptic conditions is marked green and the area of patients with infection is marked pink. (**C)** Distribution of the immune cell population in particular clusters. Y axis shows the average values of the used markers/cell counts normalised to the maximum value in the data set. (**D)** Distribution of parameters used for network construction. Areas of patients with aseptic conditions and infection are marked by a grey contour. (**E)** Distribution of neutrophils (NEUs), concentration of α-defensin, total CD45^+^ count and infection according to the EBJIS 2021 definition. **F** Distribution parameters used for network construction between clusters. For numerical values of particular immune cell populations, 95% confidence intervals and P-values, see Supplementary Table S4 and Supplementary Table S5. Legend: ↑↑↑ highest, ↑↑ moderate, ↑ lowest percentage/MFI expression of markers or cell populations across the network

Next, we performed multivariate unsupervised PSN analysis based on the similarities in the cellular and soluble markers in patients with PJI-all and OL/AL. The best clustering was achieved using a combination of six parameters: percentages of cDC2 cells from CD11b^+^ MON-Mϕ, LYM, NK cells from LYM, HLA-DR^+^ NK cells and CD88^+^ cDC1 cells and the activation of HLA-DR^+^ CD11b^+^ MON–Mϕ cells. This divided patients into four clusters, of which cluster 1 (C1) and C3 included predominantly patients with OL/AL and clusters C2 and C4, patients with PJI (Fig. 2). Cellular patterns associated with PJI, including low-grade PJI, were as follows: a low percentage of cDC2 cells from CD11b^+^ MON-Mϕ, a high percentage of CD88^+^ cDC1 cells (evident mainly in patients with positive microbiology), low LYM with a high proportion of NK cells and a low percentage of HLA-DR^+^ NK cells, as well as low expression of HLA-DR on CD11b^+^ MON–Mϕ cells. For the distribution of other parameters in individual clusters, see Fig. 2, Supplementary Table S4 and Table S5. Cluster C1, characteristic by more pronounced adaptive immune profile and higher expression of HLA-DR by CD4^+^ and CD8^+^ T-LYM, NK cells, and CD11^+^ MON–Mϕ lineage cells, also included patients with PJI with positive microbiology scored as ‘infection unlikely’ (McNally et al. 2021) located at the cluster boundary (low eccentricity) and differing from patients with OL/AL by a high percentage of CD88^+^ cDC1 cells. The cut-off values obtained from the ROC and combined ROC model curves for individual and multiple parameters used for PSN construction are listed in the Supplementary Fig. S4 and Supplementary Table S6.

### Assessment of interconnectivity between infection- and wear particle-associated mechanisms

**Fig. 3:**
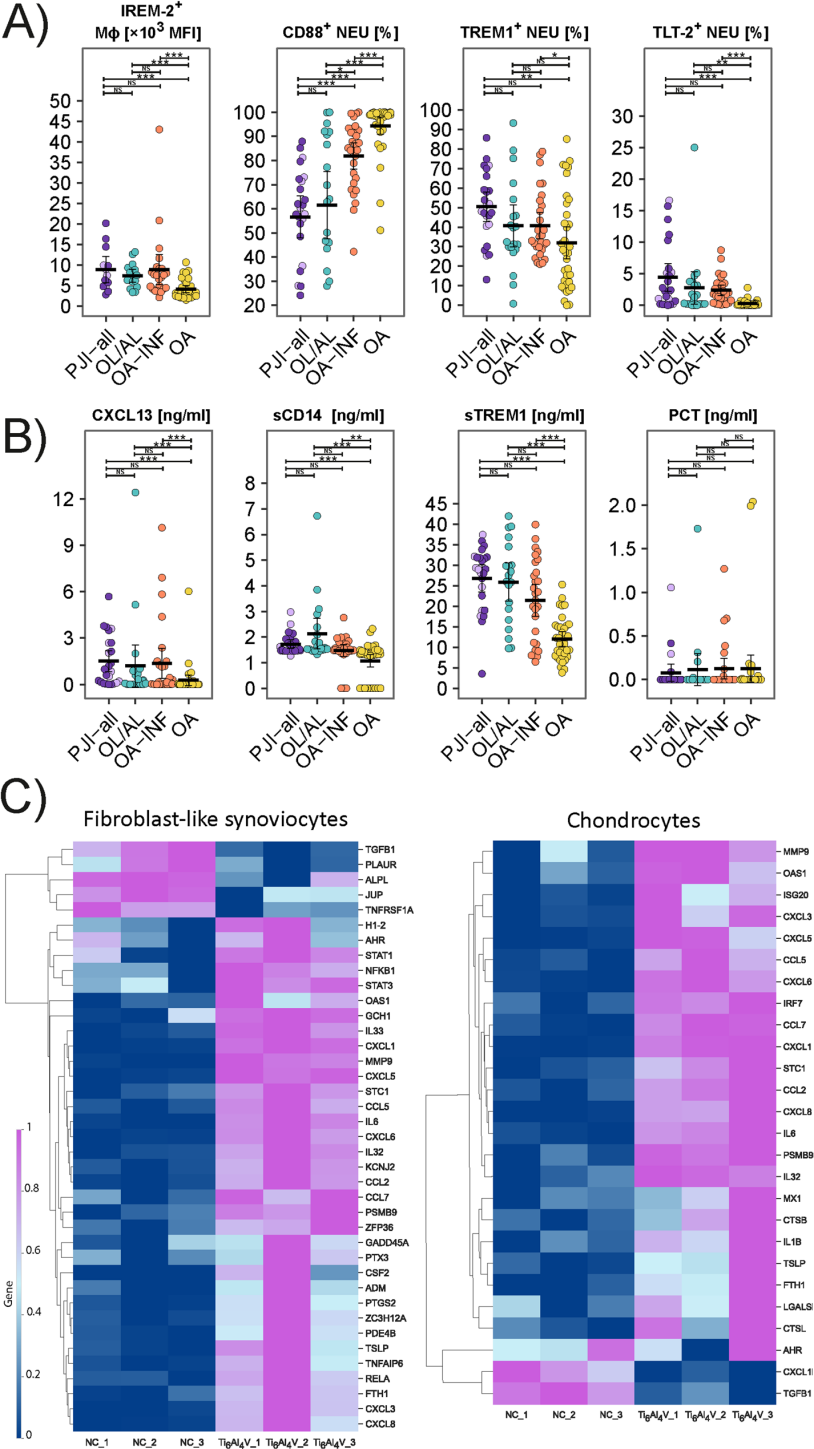
Assessment of interconnectivity between infection- and wear particle-associated mechanisms by (**A**) immunophenotypes of cells in synovial fluid in patients with/without infection and (**B**) soluble mediator patterns in synovial fluid; (**C**) expression profiling of inflammatory-related molecules in primary cell lines of fibroblast-like synoviocytes and chondrocytes from patients with OA, cultured with/without Ti_6_Al_4_V wear particles (Ti_6_Al_4_V1-V3/NT1-NT3) in three biological replicates. Full list of differentially expressed genes can be found in Supplementary Table S7. Horizontal bar represents mean, and whiskers represent 95% CI. P-values are represented as follows: *** *p* ≤ 0.001; ** 0.001 < *p* ≤ 0.01 and * 0.01 < *p* ≤ 0.05. Legend: PJI-all = patients with microbiologically confirmed prosthetic joint infection (PJI; dark violet) and with low-grade PJI (light violet); OL/AL = patients with osteolysis or aseptic loosening of the implant; OA-INF = patients with osteoarthritis and joint infection; OA = patients with osteoarthritis; NS = no significance

Next, we cultured the primary human FLSs and chondrocytes derived from OA with and without Ti_6_Al_4_V alloy particles and analysed them using RNAseq. Higher gene expression of the pro-inflammatory mediators *IL6, CXCL1/3/5/6/8, CCL2/7* and *MMP9* and lower expression of *TGFB1* was observed in both cell lines treated with Ti_6_Al_4_V alloy particles than in the untreated controls (Fig. 3). In stimulated FLS cells, higher expression of *NFKB1* and *RELA* was observed, further confirming the involvement of the NF-κB pathway in the wear particle-associated mechanism. The FLS cells, after Ti_6_Al_4_V treatment, expressed higher levels of *PTX3*, a candidate marker of PJI. For a list of differentially expressed inflammation-related genes, see Supplementary Table S7.

### Immunophenotypes of synovial fluid-derived cells and soluble mediators associated with low-grade prosthetic joint infection

**Fig. 4:**
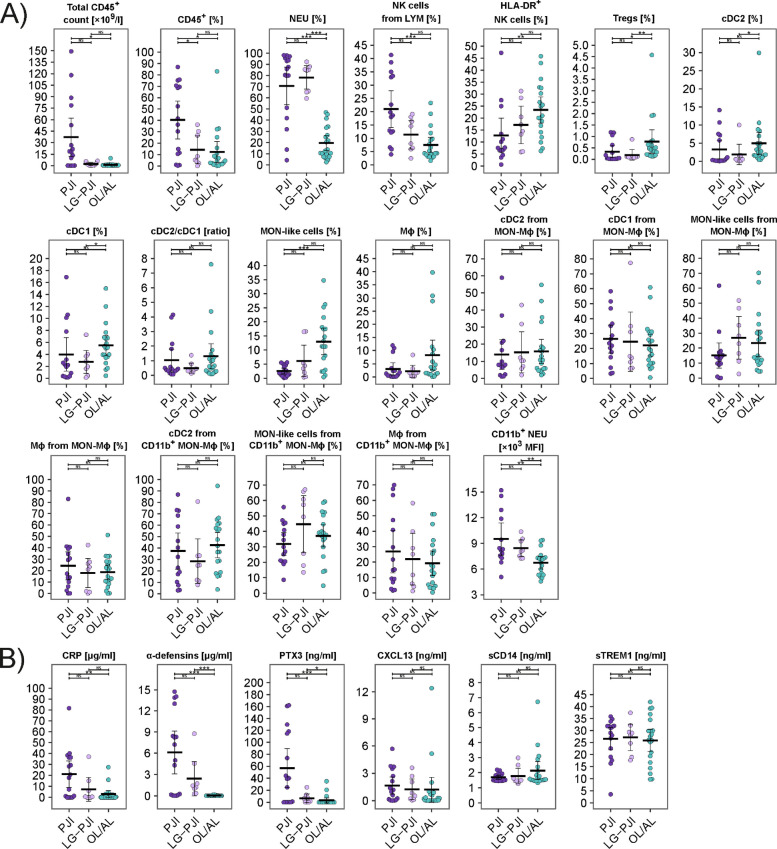
Distribution of cellular and soluble biomarkers in synovial fluid in patients with prosthetic joint infection (PJI), low-grade PJI (LG-PJI) and the control group with aseptic loosening (OL/AL). (A) Immunophenotypes of synovial fluid-derived immune cells. (B) Concentration of soluble mediators in synovial fluid. Horizontal bar represents mean, and whiskers represent 95% CI. P-values are represented as follows: *** *p* ≤ 0.001; ** 0.001 < *p* ≤ 0.01 and * 0.01 < *p* ≤ 0.05. Legend: PJI = patients with microbiologically confirmed prosthetic joint infection; LG-PJI = patients with low-grade PJI; OL/AL = patients with osteolysis or aseptic loosening of the implant; NS = no significance.*** *p* ≤ 0.001; ** 0.001 < *p* ≤ 0.01 and * 0.01 < *p* ≤ 0.05

**Fig. 5:**
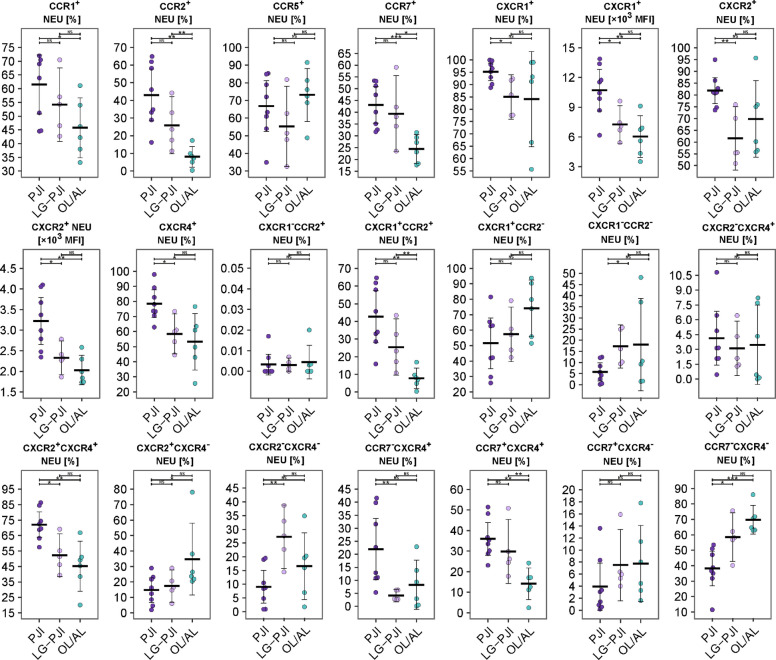
Expression of chemokine receptors on synovial fluid-derived neutrophils in patients with total joint arthroplasty with prosthetic joint infection with positive culture (PJI), low-grade PJI (LG-PJI) and aseptic loosening (OL/AL). Horizontal bar represents mean, and whiskers represent 95% CI. P-values are represented as follows: *** *p* ≤ 0.001; ** 0.001 < *p* ≤ 0.01 and * 0.01 < *p* ≤ 0.05. Legend: PJI = patients with microbiologically confirmed prosthetic joint infection; LG-PJI = patients with low-grade PJI; OL/AL = patients with osteolysis or aseptic loosening of the implant; NS = no significance

Comparing PJI, low-grade PJI and OL/AL, the total CD45^+^ cell counts and percentage of CD45^+^ cells from SF were higher in the PJI group than in the low-grade PJI and OL/AL groups (Fig. 4). Percentages of NEUs and their CD11b expression, percentage of cDC2 cells from CD45^+^ cells, and Tregs did not differ between low-grade PJI and PJI. In low-grade PJI and OL/AL, percentages of cDC2, MON-like and Mϕ were lower than in OL/AL, showing an increasing trend: PJI < low-grade PJI < OL/AL. In low-grade PJI and OL/AL, a higher percentage of NK cells, which had a low level of activation as assessed by the low percentage of HLA-DR^+^ NK cells, was detected with a decreasing trend: PJI > low-grade PJI > OL/AL. The highest percentage of CXCR4^+^ and double-positive CXCR1^+^CCR2^+^, CXCR2^+^CXCR4^+^ and CCR7^+^CXCR4^+^ NEUs and higher expression of CXCR1 and CXCR2 on NEUs were associated with PJI, with a decreasing trend: PJI > low-grade PJI > OL/AL (Fig. 5). For similarities in NEU immunophenotypes and other cells in SF between PJI and low-grade PJI, see Supplementary data, Fig. S5, Fig. S6, Table S8 and Table S9.

Regarding soluble markers, patients with low-grade PJI had lower levels of α-defensins, CRP and PTX3 in SF than those with PJI but higher levels than those with OL/AL (Fig. 4). There were no differences in CXCL13, sTREM1, sCD14 or PCT levels between low-grade PJI, PJI and OL/AL (Fig. 4).

## Discussion

Despite recent advances in diagnostics of PJI, low-grade PJI remains difficult to diagnose. In this study, we present a multiparametric approach based on the immunophenotyping of SF-derived immune cells, which may enhance diagnostic accuracy. Moreover, we demonstrated that wear particles may discriminate some biomarkers of PJI as they activate inflammatory responses mimicking infectious conditions.

Here, we comprehensively investigated the cellular content and immunophenotypes of SF-derived immune cells as candidate biomarkers for PJI detection and compared them with the soluble infection-associated biomarkers and stratification according to the EBJIS criteria combining clinical evaluation together with an analysis of serum, SF and tissues from the most suspicious areas (McNally et al. 2021). In PJI-all and OA-INF, higher immune cell counts, higher percentages of NEUs, larger EOS-like subpopulations and lower percentages of MON–Mϕ and LYM were detected than in OL/AL and OA. Generally, NEUs are the major players in defence against invading pathogens (Aroca-Crevillen et al. 2024). EOSs are also known for their defence against infections (Klion et al. 2020), however, their role in PJI deserves further investigation.

Next, we applied multivariate unsupervised network analysis to synovial effusion immune cell distribution and phenotype and revealed a composite cellular signature associated with PJI, including low-grade PJI. Specifically, PJI clustered with low percentages of cDC2 and HLA-DR^+^ NK cells, low LYM with a higher proportion of NK cells, a higher percentage of CD88^+^ cDC1 cells and the activation of CD11b^+^ MON–Mϕ cells are associated with PJI, including low-grade PJI. In addition, the cellular profile associated with PJI showed reduced HLA-DR expression on CD4^+^ and CD8^+^ T-LYM, NK cells, and CD11^+^ MON–Mϕ lineage cells. In summary, these findings suggest that SF-derived immune cell pattern information may complement established diagnostic workups by capturing PJI-associated immune dysregulation even in the presence of mild inflammation around the implant, thereby supporting diagnostics of challenging cases such as low-grade PJI. From a immunological point of view, the immune pattern associated with PJI is characterized by suppression of adaptive immunity along with activation of innate immunity, probably caused by a ‘dysregulated host response’, as already described in critical infections and sepsis (Zhou et al. 2021; Venet and Monneret 2018; Cajander et al. 2024). Future work should determine whether these synovial immune signatures are transient or persistent and whether they relate to clinically relevant outcomes such as persistence or recurrence.

Among the most important parameters associated with PJI were low cDC2, a subset of MON–Mϕ lineage cells present in abundance in OA knees (Mikulkova et al. 2024), and a lower percentage of HLA-DR^+^ NK cells, a subset of NK cells with the phenotypic characteristics of both NK cells and DCs (Erokhina et al. 2021). The depletion of cDC2 is recognised as an early event during systemic bacterial infection (Bieber et al. 2021) as well as viral infections, as shown by SARS-CoV-2 infection (Chang et al. 2022). Given that cDC2 play a key role in promoting the innate immune response, they influence a wide range of T-LYM responses, and are important producers of IL-23, IL-1, TNF-α, IL-8, and IL-10 (Leon 2025), their depletion may contribute to the impaired immune response and the severity of infection also in our patients. Therefore, further research on the role and functional heterogeneity of SF-derived cDC2s is warranted. In addition, PJI was characterised by elevated proportion of NK cells able to kill bacteria, fungi and parasites as well as virus-infected cells (Vivier et al. 2024; Thomas and Yang 2016). Additionally, high representation of CD88^+^ cDC1, a subset of DCs with enhanced antigen cross-presentation capacities (Si et al. 2023) was evident, particularly in PJI samples with positive microbiology. Our data showed that the assessment of immune cell phenotypes might have effective diagnostic performance for low-grade PJI and culture-negative PJI, as also shown by others (Li et al. 2023). Moreover, our study highlights the need to use a combination of different biomarkers rather than a single biomarker to improve cumulative diagnostic accuracy in PJI.

Next, we were interested in the deep characterisation of NEUs in PJI and low-grade PJI. Of the studied chemokine receptors, the highest percentage of CCR1/2/7^+^ populations, associated with the inflammatory phenotype (Capucetti et al. 2020), CXCR2^+^CXCR4^+^ and double-positive CXCR1^+^CCR2^+^ and CXCR2^+^CXCR4^+^populations, involved in mobilisation during inflammation and clearance (Martin et al. 2003), and higher expression of pro-inflammatory CXCR1 and CXCR2 on NEUs was detected in PJI, with a decreasing trend: PJI > low-grade PJI > OL/AL. The majority of NEUs in PJI with culture positivity are CXCR4^+^, which suggests that these NEUs may be exhausted and provide ongoing CXCR4‐dependent NEU clearance, as shown in animal models (Martin et al. 2003). Another animal model study reported a contribution of CXCR4^+^ NEUs to tissue damage during bacterial infection (Gawish et al. 2022). By contrast, NEUs in low-grade PJI expressed less CXCR1, CXCR2 and CXCR4 than in PJI, and all chemokine receptors were associated with a less pronounced antimicrobial phenotype and less exhaustion of NEUs. In addition, a high percentage of CXCR2^−^CXCR4^−^ NEUs, a subset of constitutively mobilised NEUs identified in a murine model (Eash et al. 2010), was characteristic for low-grade PJI. Our data on different subsets of NEUs in PJI and low-grade PJI deserves further investigation.

Furthermore, we examined the overlap between infection- and wear particle-driven pathways (Gibon et al. 2024), because both can amplify local inflammation and thereby distort the selection of PJI biomarkers. Consistent with this, CXCL13, sCD14, and sTREM1 reached similarly high levels in SF from PJI and OL/AL, which limits their utility for discriminating infection from aseptic failure. To gain more insight into the changes induced by wear particles, we stimulated OA fibroblast-like synoviocytes that are directly exposed to intra-articular debris and contribute to chronic inflammation, and OA chondrocytes, which can also respond to prosthetic particles (Bauer et al. 2021), with Ti_6_Al_4_V alloy. In both cell types, Ti_6_Al_4_V stimulation increased expression of mediators commonly linked to infection and inflammation (*IL6*, *IL1B*, *MMP9*, *PTX3, CCL2*, *CXCL1/3/6/8)*, as well as NF-κB pathway (*RELA* and *NFKB)*, supporting the interconnectivity between infection-related mechanisms and wear-related inflammation, as also reported by others (Koreny et al. 2006; Tunyogi-Csapo et al. 2008; Wu et al. 2020). This transcriptomic response was mirrored by the shared pro-inflammatory immunophenotype of SF-derived immune cells from PJI and OL/AL. We used larger titanium particles (15–45 μm), which are less cytotoxic than smaller particles yet remain immunologically active (Callejas et al. 2022). By contrast, nano-sized particles (< 1 μm) are predicted to elicit strong pro-inflammatory responses due to higher bioactivity and efficient phagocytic uptake (Callejas et al. 2022; Zhang et al. 2024; Yao et al. 2017). Since nano-sized particles constitute the majority of wear debris in the tissues surrounding implants (Maloney et al. 1995), we may expect an even more pronounced pro-inflammatory response, higher cytotoxicity and other adverse effects (Maloney et al. 1995; Keeney et al. 2013; Ganko et al. 2025) on the cells surrounding the implants compared to our model of mechanical irritation of cells by larger titanium particles. In addition, titanium particles have been also shown to increase cytokine production in human osteoblasts (Costa et al. 2019) and to activate NEUs, including enhanced respiratory burst, NEU extracellular trap formation, and IL-6 release (Kalra 2013). More broadly, accumulating evidence indicates that wear/corrosive particles from other implant materials and additional cell types can elicit similar inflammatory programs (Yao et al. 2025), further reducing specificity of some biomarkers. Likewise, cobalt–chromium–molybdenum (CoCrMo) particles induce inflammatory activation of macrophages and other phagocytes, promoting production of reactive oxygen species, matrix metalloproteinases, IL-6, TNF-α, and other mediators (Pearson et al. 2015; Wan et al. 2008; Cai et al. 2025). Clinically, metallosis and polyethylene wear may also trigger α-defensin production and yield false-positive test results (Bonanzinga et al. 2019). Together, these observations underscore why immune-related biomarkers such as MMP9 (Li et al. 2024), PTX3 (Loppini et al. 2023), or IL-6 (Li et al. 2022) can be strongly influenced by wear particle-driven inflammation and, therefore, require careful validation in cohorts enriched for aseptic particle disease as well as low-grade PJI.

This study has several limitations. The low-grade PJI subgroup was relatively small and reflects the actual distribution of PJI phenotypes: as inflammatory activity decreases, diagnostic uncertainty increases, fewer cases meet stringent criteria at the time of work-up, and some infections remain unrecognised or are managed as ‘aseptic’ revisions (Ince et al. 2004; Perez-Prieto et al. 2017; Trebse and Roskar 2021) Importantly, despite its size, the low-grade PJI subgroup represents a clinically relevant population in whom current diagnostic approaches perform least reliably. Next, our analyses captured a single aspiration time point, which limits generalisability across different stages of PJI and across non-implant-related joint infections. Moreover, clinical diagnosis of low-grade PJI remains imperfect even when contemporary criteria are applied. Although we used the current EBJIS definition (McNally et al. 2021), diagnostic uncertainty is inherent in low-grade presentations and may have led to misclassification within the cohort. Other frameworks (e.g., W.A.I.O.T.) might label some EBJIS ‘infection confirmed’ or ‘infection likely’ cases as ‘biofilm-related’ (Romano et al. 2019); however, these categories are not yet sufficiently validated to serve as a robust reference standard. The in vitro model of particle-induced inflammation was deliberately simplified: we tested only two cell types- commercial cell lines each derived from one patient with OA and Ti_6_Al_4_V particles within a defined size range. Responses may differ with other clinically relevant wear materials, particle characteristics, and joint-resident cell populations. We did not investigate specific inflammatory, cytotoxic, hypersensitivity, or osteolysis-related signalling pathways; therefore, future studies should address the underlying specific and nonspecific mechanisms of wear particle–induced cellular effects. Despite a comprehensive panel of immune and soluble markers was used, additional markers may be associated with PJI and should be explored in future work. Finally, future studies should determine whether the immune dysregulation observed in SF can be captured using less invasive sampling, such as peripheral blood, and whether it has prognostic implications.

Taken together, this study introduces a novel host-oriented strategy for PJI detection based on SF-derived immune cells and their immunophenotyping, offering an advanced diagnostic approach for PJI, including low-grade PJI. By shifting the emphasis from pathogen detection and ambiguous clinical manifestations to immune profiling, this approach captures the complex host immune response to the infection stimuli. Further, this approach improves diagnostic accuracy by distinguishing infection from sterile inflammation induced by wear particles -thereby reducing the number of false positive and false negative results. Our findings provide a mechanistic basis for refining current diagnostic algorithms and support the incorporation of host immune features into the routine evaluation of suspected PJI.

## Supplementary Information

Supplementary Material 1.

## Data Availability

All data are available upon reasonable request.
