# Supplementary material for: A novel host-based strategy for detecting low-grade prosthetic joint infection using immune cell pattern in synovial fluid
Source: Mol Med. 2026 Mar 28;32:71. doi: 10.1186/s10020-026-01461-0 (PMC13151374; doi:10.1186/s10020-026-01461-0)
Supplement: Supplementary file 1 — Supplementary Material 1. [file 10020_2026_1461_MOESM1_ESM.pdf]

SUPPLEMENTARY DATA

A novel host-based strategy for detecting low-grade prosthetic joint infection using immune cell pattern in synovial fluid

Trajerova M, Kriegova E, Shrestha B, Kudelka M, Savara J, Gallo J

RESULTS

Patient similarity network identified the cellular pattern associated with low-grade prosthetic joint infection

A multivariate patient similarity network (PSN) based on the similarities in the cellular pattern and surface markers on neutrophils (NEUs) was utilised to detect the patterns associated with low-grade prosthetic joint infection (PJI). Among the parameters used, the best clustering of patients was possible using percentages of natural killer (NK) cells from lymphocytes (LYMs), dendritic cell type cDC2, CD88^{+} cDC1 cells, TERM1^{+} cDC1, monocyte (MON)-like cells, NEUs, TREM1^{+} NEUs, the percentage of double-negative CCR7^{-}CXCR4^{-} and CXCR2^{-}CXCR4^{-} NEU populations and TLT-2^{+} CD8^{+} T-LYM, together with the expression (determined through median fluorescence intensity (MFI)) of CXCR1 on NEUs and IREM-2 on macrophages (**Supplementary Figure S3A**). Cluster 2 and cluster 4 contained mostly PJI samples, and cluster 3 contained aseptic OL/AL samples (**Supplementary Figure S3B**). Low-grade PJI samples shared cluster 1 with aseptic conditions and cluster 2 with PJI samples. Of the network building parameters (**Supplementary Figure S3C, S3D, S3E**), three coincided with the PSN used to distinguish PJI groups from OL/AL, which were: cDC2 from CD11b^{+} MON-Mϕ (%), CD88^{+} cDC1 (%), NK from LYM (%). Cluster 2 shows characteristics typical for infection samples (**Supplementary Figure S3F**): a high immune cell count, a high percentage of NEUs and high expression of the inflammatory receptors CCR2 and CXCR4 and the soluble mediators CRP, α-defensins and PTX3. The parameters of cluster 4 were the same as those of cluster 2 but with less intensity. Patients in both clusters showed a shortened time from the start of joint difficulties. However, cluster 1 and, especially, cluster 3 contained patients with a longer time since the start of joint difficulties. Both these clusters showed low levels of the aforementioned markers. The difference between these clusters is the percentage of NEUs, which is higher in cluster 1. The full distribution of synovial fluid (SF)-derived immune cells and their activation are detailed in **Supplementary Table S7.** An overview of P-values can be found in **Supplementary Table S8.**

Supplementary Table S1. Characterisation of individual patients with total joint arthroplasty according to the synovial fluid (SF)-related criteria linked to the definition of prosthetic joint infection according EBJIS[1].

Patient ID	Group	Localization of arthroplasty	α-defensins [ng/mL]		PMN [%]			$CD45^+$ cellularity [$*10^9$/l]			CRP [µg/ml]		Detected pathogen	Synovial fluid-related criteria of PJI according EBJIS	Criteria of PJI according EBJIS
			<1,560	≥1,560	<65	65-80	≥80	<1.5	1.5–3.0	≥3.0	<6.9	≥6.9			
1	OL/AL	knee	X		X			X			X		negative	Infection unlikely	no
2	OL/AL	knee	X		X			X				X	negative	Infection unlikely	no
3	OL/AL	knee	X		X			X			X		negative	Infection unlikely	no
4	OL/AL	knee	X		X			X			X		negative	Infection unlikely	no
5	OL/AL	knee	X		X				X		X		negative	Infection unlikely	no
6	OL/AL	knee	X		X			X			X		negative	Infection unlikely	no
7	OL/AL	knee	X		X				X		X		negative	Infection unlikely	no
8	OL/AL	hip	X		X			X			X		negative	Infection unlikely	no
9	OL/AL	hip	X		X				X			X	negative	Infection unlikely	no
10	OL/AL	hip	X		X			X			X		negative	Infection unlikely	no
11	OL/AL	knee	X		X			X			X		negative	Infection unlikely	no
12	OL/AL	hip	X		X					X	X		negative	Infection confirmed	no
13	OL/AL	hip	X		X			X			X		negative	Infection unlikely:	no
14	OL/AL	knee	X		X			X			X		negative	Infection unlikely:	no
15	OL/AL	knee	X		X			X			X		negative	Infection unlikely:	no
16	OL/AL	knee	X		X			X			X		negative	Infection unlikely:	no
17	OL/AL	knee	X		X			X			X		negative	Infection unlikely:	no
18	OL/AL	hip	X		X			X			X		negative	Infection unlikely:	no
19	OL/AL	knee	X		X			X			X		negative	Infection unlikely:	no
20	OL/AL	hip	X		X			X			X		negative	Infection unlikely:	no
21	LG-PJI	knee		X			X			X		X	negative	Infection confirmed	yes
22	LG-PJI	knee	X				X	X			X		negative	Infection confirmed	yes
23	LG-PJI	knee		X		X		X			X		negative	Infection confirmed	yes
24	LG-PJI	knee	X		X				X		X		negative	Infection unlikely	yes
25	LG-PJI	hip	X				X	X			X		negative	Infection confirmed	yes
26	LG-PJI	knee		X			X			X		X	negative	Infection confirmed	yes
27	LG-PJI	knee	X				X		X		X		negative	Infection confirmed	yes
28	LG-PJI	knee	X			X		X			X		negative	Infection unlikely	yes
29	PJI	knee		X			X			X		X	*Staphylococcus aures*	Infection confirmed	yes
30	PJI	knee	X				X			X		X	*Staphylococcus aureus*	Infection confirmed	yes
31	PJI	hip		X			X			X		X	*Streptococcus intermedius*	Infection confirmed	yes
32	PJI	knee		X			X			X		X	*Staphylococcus epidermidis*	Infection confirmed	yes
33	PJI	knee		X			X			X		X	*Staphylococcus epidermidis*	Infection confirmed	yes
34	PJI	knee		X			X			X		X	*Streptococcus salivarius*	Infection confirmed	yes
35	PJI	knee		X		X				X		X	*Streptococcus mitis*	Infection confirmed	yes
36	PJI	knee		X			X			X		X	*Listeria monocytogenes*	Infection confirmed	yes
37	PJI	hip	X		X			X			X		*Staphylococcus hominis*	Infection unlikely	yes
38	PJI	knee		X		X				X	X		*Parvimonas micra*	Infection confirmed	yes
39	PJI	knee	X		X			X			X		*Staphylococcus caprae*	Infection unlikely	yes

40	PJI	hip	X		X			X			X		*Staphylococcus epidermidis a Staphylococcus hominis*	Infection unlikely	yes
41	PJI	knee	X		X			X			X		*Staphylococcus aureus*	Infection unlikely	yes
42	PJI	knee		X			X			X		X	*Streptococus agalactiae*	Infection confirmed	yes
43	PJI	knee		X			X			X		X	*Staphylococcus epidermidis*	Infection confirmed	yes
44	PJI	knee	X		X			X			X		*Staphylococus warneri, Acinetobacter lwofii*	Infection unlikely	yes

Legend: NEU: neutrophils; **OL/AL:** osteolysis/aseptic loosening; **LG-PJI:** low-grade prosthetic joint infection; **PJI:** microbiologically confirmed prosthetic joint infection**.**

Supplementary Table S2. List of antibodies used for flow cytometry analysis of synovial fluid-derived immune cells.

Marker	Clone	Fluorophore	Cat. No.	Vendor	RRID_ID	Panel
CCR2	K036C2	AF488	357226	BioLegend	AB_2650961	3
IgG2a	MOPC-173	AF488	400233	BioLegend	AB_3076443	6
CD11b	ICRF44	AF700	301356	BioLegend	AB_2750075	1+2+3
IgG1	MOPC-21	AF700	400144	BioLegend	AB_2923250	4+5+6
CCR5	J418F1	APC	359122	BioLegend	AB_2564073	3
CD88	S5/1	APC	344310	BioLegend	AB_11204420	2
HLA-DR	L243	APC	307610	BioLegend	AB_314688	1
IgG2a	MOPC-173	APC	400222	BioLegend	AB_2891178	4+5
IgG2b	MPC-11	APC	400321	BioLegend	AB_326500	6
CD14	HCD14	APC-Cy7	325620	BioLegend	AB_830693	2
CD62L	DREG-56	APC-Cy7	304814	BioLegend	AB_493582	3
CD64	10.1	APC-Cy7	305026	BioLegend	AB_2561588	1
IgG1	MOPC-21	APC-Cy7	400128	BioLegend	AB_2892538	4+5+6
CD25	M-A251	BV421	356114	BioLegend	AB_2562164	1
CD45	HI30	BV421	563879	BD Biosciences	AB_2744402	3
TLT2	MIH61	BV421	744882	BD Biosciences	AB_2742559	2
IgG1	MOPC-21	BV421	400158	BioLegend	AB_11150232	4+5+6
CCR7	G043H7	BV510	353232	BioLegend	AB_2563866	3
CD45	HI30	BV510	563204	BD Biosciences	AB_2738067	1
HLA-DR	L243	BV510	307646	BioLegend	AB_2561948	2
IgG1	MOPC-21	BV510	400172	BioLegend	AB_2714004	4
IgG2a	MOPC-173	BV510	400267	BioLegend	AB_2734131	5+6
CD15	W6D3	BV605	323032	BioLegend	AB_2562132	1+3
TREM1 (CD354)	6B1	BV605	743740	BD Biosciences	AB_2741712	2
IgG1	MOPC-21	BV605	400162	BioLegend	AB_11125373	4+5+6
CD16	3G8	BV650	302042	BioLegend	AB_2563801	3
CD4	RPA-T4	BV650	300536	BioLegend	AB_2632791	1+2
IgG1	MOPC-21	BV650	400164	BioLegend	AB_11126986	4+5+6
CD14	M5E2	BV711	301838	BioLegend	AB_2562909	1+3
IREM-2 (CD300e)	UP-H1	BV711	745476	BD Biosciences	AB_2743014	2
IgG2a	MOPC-173	BV711	400272	BioLegend	AB_3097679	4+5+6
CD3	OKT3	BV785	317330	BioLegend	AB_2563507	3
CD8	SK1	BV785	344740	BioLegend	AB_2566202	1+2
IgG1	MOPC-21	BV785	400169	BioLegend	AB_11219601	4+5
IgG2a	MOPC-173	BV785	400274	BioLegend	AB_2923257	6
IgG1	MOPC-21	FITC	400110	BioLegend	AB_2861401	4+5
CD3/CD16+56	UCHT1/3G8 +MEM-188	FITC/PE	319101	BioLegend	AB_314999	1+2
CCR1	5F10B29	PE	362904	BioLegend	AB_2563898	3
CD45	2D1	PE	368510	BioLegend	AB_2566370	*native count*
IgG1	MOPC-21	PE	400114	BioLegend	AB_326435	4+5+6
IgG2a	MOPC-173	PE	400213	BioLegend	AB_2800438	4+5
CD45RO	UCHL1	PE/Dazzle594	304248	BioLegend	AB_2566543	2
CXCR4	12G5	PE/Dazzle594	306526	BioLegend	AB_2564065	3
IgG2a	MOPC-173	PE/Dazzle594	400275	BioLegend	AB_3097694	5+6
CD15	W6D3	PE-Cy7	323030	BioLegend	AB_2561670	2
CXCR2	5E8/CXCR2	PE-Cy7	320716	BioLegend	AB_2564597	3
IgG1	MOPC-21	PE-Cy7	400126	BioLegend	AB_326448	5+6
CD127	A019D5	PerCP-Cy5.5	351322	BioLegend	AB_10897104	1
CD45	HI30	PerCP-Cy5.5	564105	BD Biosciences	AB_2744405	2
CXCR1	8F1/CXCR1	PerCP-Cy5.5	320622	BioLegend	AB_2565552	3
IgG1	MOPC-21	PerCP-Cy5.5	400150	BioLegend	AB_893664	4+5
IgG2b	MPC-11	PerCP-Cy5.5	400337	BioLegend	AB_2937017	6

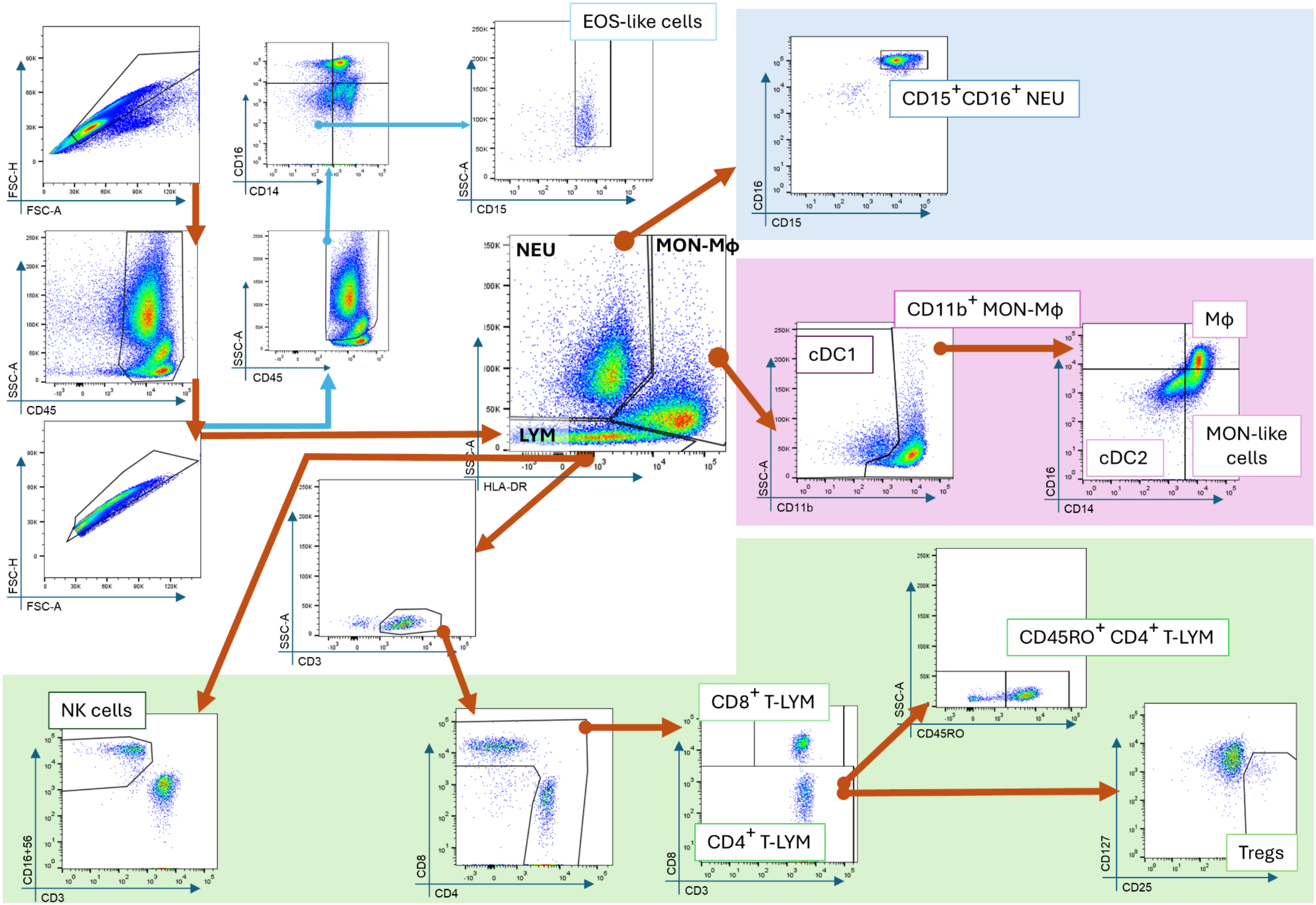

Supplementary Figure S1. Gating strategy for synovial fluid-derived immune cells in synovial fluid in KOA.

Legend: LYM: lymphocytes, Mon-Mϕ: monocyte-macrophage lineage cells; NEUs: neutrophils; EOS-like: eosinophil-like cells; Mϕ: macrophage; MON-like: monocyte-like cells; cDC1: conventional dendritic cells type 1; cDC2: conventional dendritic cells type 2; NK cells: natural killer cells; CD8$^+$ T-LYM: cytotoxic T-lymphocytes; CD4$^+$ T-LYM: helper T-lymphocytes; Tregs: regulatory T-lymphocytes.

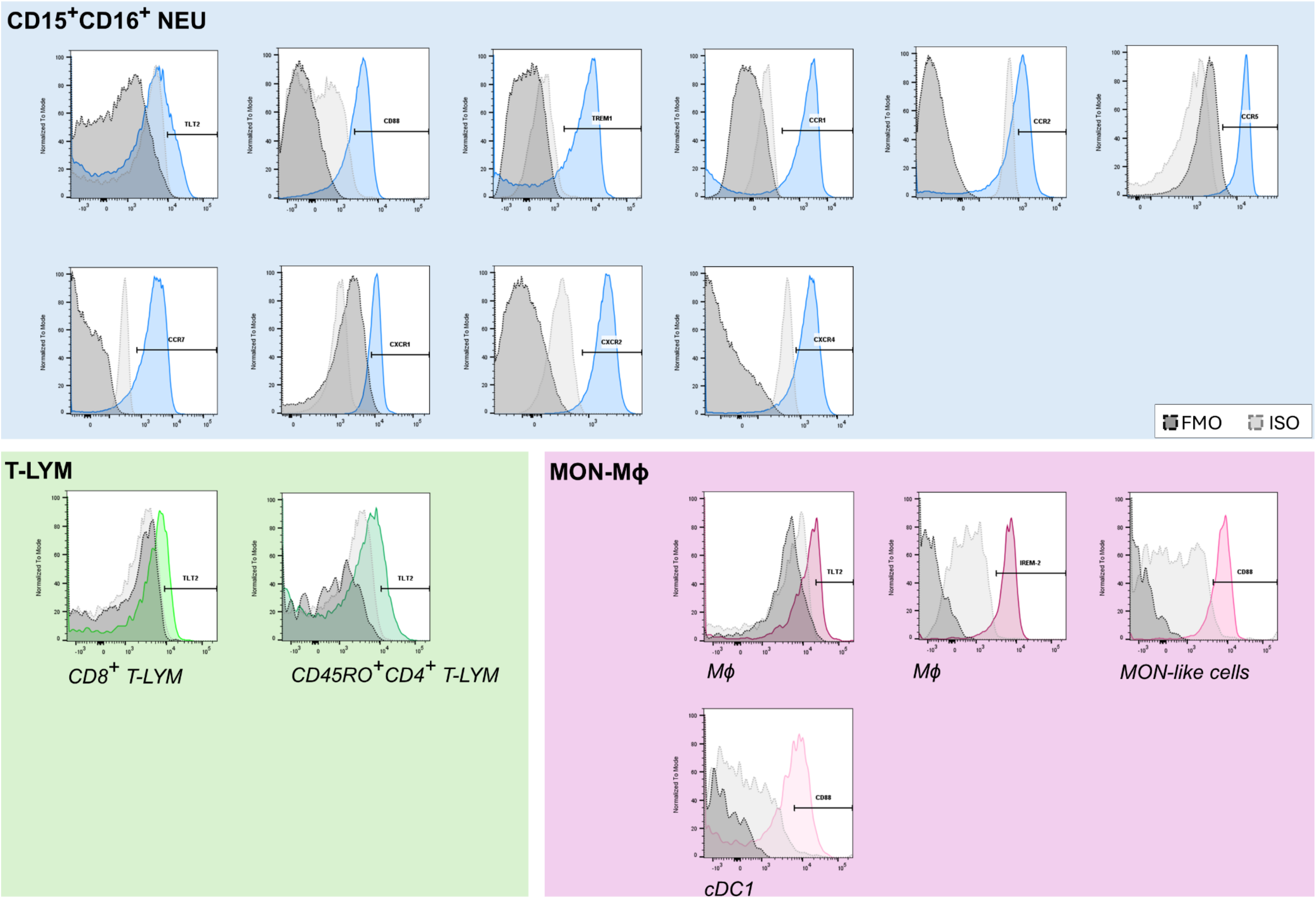

Supplementary Figure S2. Expression of analysed markers on synovial-fluid-derived immune cells in knee/hip osteoarthritis/total joint arthroplasty.
Legend: FMO: fluorescence minus one control; ISO: isotype control; T-LYM: T-lymphocytes, MON-Mϕ: monocyte-macrophage lineage; NEUs: neutrophils; Mϕ: macrophage; MON-like: monocyte-like cells; cDC1: conventional dendritic cells type 1; $CD8^+$ T-LYM: cytotoxic T-lymphocytes; $CD4^+$ T-LYM: helper T-lymphocytes.

Supplementary Table S3: Detailed characterization of patient groups.

	PJI-all	LG-PJI	PJI	OL/AL	OA-INF	OA	P-value<br>PJI-all vs OL/Al vs OA-INF vs OA	P-value<br>PJI vs LG-PJI vs OL/AL
**No of patients**	**24**	8	16	**20**	**28**	**36**		
**Men/women**	**17/7**	7/1	10/6	**12/8**	**13/15**	**24/12**		
**Knee/hip**	**20/4**	7/1	13/3	**13/7**	**28/0**	**36/0**		
Total $CD45^+$ count [$\times 10^9$/l]:<br>**mean** ± 95% CI; [min–max]; *NA*	**25.6** ± 17.5;<br>[0.1–149.1]; *0*	**37.3** ± 24.9;<br>[0.1-149.1]; *0*	**2.4** ± 2.0;<br>[0.5-6.3]; *0*	**1.2** ± 1.0;<br>[0.1–9.7]; *0*	**17.3** ± 6.4;<br>[0.2–59.3]; *0*	**0.5** ± 0.2;<br>[0.1–2.9]; *0*	**<0.001**	**0.031**
Percentage of $CD45^+$ cells%:<br>**mean** ± 95% CI; [min–max]; *NA*	**31.7** ± 12.3;<br>[0.1–86.9]; *0*	**40.4** ± 16.6;<br>[0.3-86.9]; *0*	**14.2** ± 12.0;<br>[0.1-36.2]; *0*	**12.4** ± 9.1;<br>[0.1–83.1]; *0*	**72.8** ± 9.6;<br>[7.0–96.3]; *0*	**29.8** ± 7.3;<br>[2.8–86.3]; *0*	**<0.001**	**0.024**
LYM [%]:<br>**mean** ± 95% CI; [min–max]; *NA*	**14.1** ± 8.3;<br>[0.5–67.4]; *0*	**17.0** ± 12.6;<br>[0.5-67.4]; *0*	**8.3** ± 3.9;<br>[3.1-15.3]; *0*	**46.5** ± 11.1;<br>[6.4–89.1]; *0*	**7.6** ± 3.2;<br>[0.2–29.5]; *0*	**34.8** ± 6.5;<br>[4.5–84.1]; *0*	**<0.001**	**<0.001**
MON-Mϕ [%]:<br>**mean** ± 95% CI; [min–max]; *NA*	**11.9** ± 4.2;<br>[0.5–28.8]; *0*	**11.5** ± 5.3;<br>[0.5-27.2]; *0*	**12.5** ± 8.7;<br>[1.0-28.8]; *0*	**32.9** ± 10.6;<br>[6.0–77.9]; *0*	**13.7** ± 5.0;<br>[0.9–66.8]; *0*	**50.5** ± 6.8;<br>[12.3–92.2]; *0*	**<0.001**	**0.002**
NEU [%]:<br>**mean** ± 95% CI; [min–max]; *NA*	**73.2** ± 11.3;<br>[4.2–98.1]; *0*	**70.7** ± 16.8;<br>[4.2-98.1]; *0*	**78.2** ± 10.5;<br>[59.4-92.2]; *0*	**19.5** ± 6.6;<br>[2.5–47.0]; *0*	**77.9** ± 7.1;<br>[1.7–97.8]; *0*	**13.6** ± 3.6;<br>[1.4–44.8]; *0*	**<0.001**	**<0.001**
EOS-like cells [%]:<br>**mean** ± 95% CI; [min–max]; *NA*	**4.8** ± 2.7;<br>[0.0–22.9]; *2*	**5.8** ± 4.0;<br>[0.0-22.9]; *2*	**3.2** ± 3.6;<br>[0.6-13.0]; *0*	**0.7** ± 0.4;<br>[0.0–2.9]; *1*	**2.0** ± 0.8;<br>[0.0–11.0]; *0*	**0.3** ± 0.2;<br>[0.0–2.5]; *0*	**<0.001**	**0.001**
NK cells [%]:<br>**mean** ± 95% CI; [min–max]; *NA*	**1.3** ± 0.8;<br>[0.1–9.1]; *1*	**1.6** ± 1.3;<br>[0.1-9.1]; *1*	**0.8** ± 0.5;<br>[0.3-2.2]; *0*	**3.4** ± 1.5;<br>[0.3–12.0]; *1*	**0.5** ± 0.3;<br>[0.0–2.9]; *0*	**4.2** ± 1.5;<br>[0.4–21.1]; *0*	**<0.001**	**0.003**
Tregs [%]:<br>**mean** ± 95% CI; [min–max]; *NA*	**0.3** ± 0.2;<br>[0.0–1.2]; *2*	**0.3** ± 0.3;<br>[0.0-1.2]; *2*	**0.2** ± 0.2;<br>[0.1-0.9]; *0*	**0.8** ± 0.5;<br>[0.1–4.6]; *1*	**0.2** ± 0.2;<br>[0.0–2.9]; *0*	**0.6** ± 0.3;<br>[0.1–4.2]; *0*	**<0.001**	**0.006**
$CD3^+$ from LYM [%]:<br>**mean** ± 95% CI; [min–max]; *NA*	**77.7** ± 5.0;<br>[53.6–92.5]; *1*	**74.4** ± 6.8;<br>[53.6-91.0]; *1*	**84.0** ± 5.6;<br>[75.9-92.5]; *0*	**85.8** ± 4.8;<br>[54.1–94.8]; *1*	**85.4** ± 2.9;<br>[65.8–94.7]; *0*	**81.9** ± 3.8;<br>[46.7–96.0]; *0*	**0.030**	**0.008**
NK cells from LYM [%]:<br>**mean** ± 95% CI; [min–max]; *NA*	**17.6** ± 5.0;<br>[2.5–41.4]; *1*	**21.0** ± 6.9;<br>[4.0-41.4]; *1*	**11.3** ± 5.3;<br>[2.5-19.1]; *0*	**7.5** ± 2.9;<br>[1.6–23.3]; *1*	**9.4** ± 2.9;<br>[1.3–29.2]; *0*	**11.9** ± 3.3;<br>[1.5–48.3]; *0*	**0.004**	**0.002**
$CD4^+$ T-LYM from $CD3^+$ [%]:<br>**mean** ± 95% CI; [min–max]; *NA*	**66.0** ± 5.7;<br>[36.5–89.8]; *2*	**67.0** ± 8.8;<br>[36.5-89.8]; *2*	**64.4** ± 6.3;<br>[53.4-71.1]; *0*	**55.1** ± 7.5;<br>[36.2–85.6]; *1*	**66.7** ± 5.9;<br>[30.4–88.1]; *0*	**44.0** ± 4.9;<br>[20.3–78.0]; *0*	**<0.001**	0.051
$CD45RO^+$ $CD4^+$ T-LYM [%]:<br>**mean** ± 95% CI; [min–max]; *NA*	**86.2** ± 8.9;<br>[0.0–99.7]; *0*	**87.7** ± 12.8;<br>[0.0-99.7]; *0*	**83.2** ± 12.1;<br>[60.5-97.6]; *0*	**90.0** ± 5.5;<br>[55.5–99.3]; *1*	**95.9** ± 1.0;<br>[90.1–99.1]; *0*	**92.1** ± 1.6;<br>[80.8–99.1]; *0*	**0.008**	0.380
$CD45RO^-$ $CD4^+$ T-LYM [%]:<br>**mean** ± 95% CI; [min–max]; *NA*	**9.5** ± 4.4;<br>[0.0–39.2]; *0*	**5.9** ± 2.8;<br>[0.0-16.4]; *0*	**16.7** ± 12.1;<br>[2.4-39.2]; *0*	**10.0** ± 5.5;<br>[0.7–44.5]; *1*	**4.1** ± 1.0;<br>[0.9–9.9]; *0*	**7.8** ± 1.6;<br>[0.9–19.1]; *0*	**0.019**	0.155
$CD8^+$ T-LYM from $CD3^+$ [%]:<br>**mean** ± 95% CI; [min–max]; *NA*	**34.0** ± 5.7;<br>[10.2–63.5]; *2*	**33.0** ± 8.8;<br>[10.2-63.5]; *2*	**35.6** ± 6.3;<br>[28.9-46.6]; *0*	**44.9** ± 7.5;<br>[14.4–63.8]; *1*	**33.3** ± 5.9;<br>[11.9–69.6]; *0*	**56.0** ± 4.9;<br>[22.0–79.7]; *0*	**<0.001**	0.051
$CD4^+$/ $CD8^+$ T-LYM [ratio]:<br>**mean** ± 95% CI; [min–max]; *NA*	**2.5** ± 0.8;<br>[0.6–8.8]; *2*	**2.8** ± 1.2;<br>[0.6-8.8]; *2*	**1.9** ± 0.5;<br>[1.1-2.5]; *0*	**1.7** ± 0.7;<br>[0.6–5.9]; *1*	**2.8** ± 0.7;<br>[0.4–7.4]; *0*	**1.0** ± 0.2;<br>[0.3–3.5]; *0*	**<0.001**	0.051
$HLA\text{-}DR^+$ $CD4^+$ T-LYM [%]:<br>**mean** ± 95% CI; [min–max]; *NA*	**22.5** ± 7.3;<br>[5.2–64.4]; *2*	**17.4** ± 6.2;<br>[5.2-46.5]; *2*	**31.3** ± 17.8;<br>[9.7-64.4]; *0*	**28.7** ± 8.3;<br>[4.7–78.3]; *1*	**35.5** ± 6.3;<br>[7.2–69.9]; *0*	**37.5** ± 6.9;<br>[5.2–90.1]; *0*	**0.003**	0.056
$HLA\text{-}DR^+$ $CD8^+$ T-LYM [%]:<br>**mean** ± 95% CI; [min–max]; *NA*	**30.2** ± 6.8;<br>[3.9–54.5]; *2*	**26.1** ± 7.7;<br>[3.9-49.2]; *2*	**37.5** ± 14.0;<br>[13.7-54.5]; *0*	**35.6** ± 10.3;<br>[8.0–83.2]; *1*	**40.5** ± 6.9;<br>[6.7–89.7]; *0*	**44.6** ± 6.3;<br>[11.4–80.8]; *0*	**0.026**	0.366
$HLA\text{-}DR^+$ NK cells [%]:<br>**mean** ± 95% CI; [min–max]; *NA*	**14.4** ± 5.1;<br>[0.6–47.3]; *2*	**12.8** ± 7.2;<br>[0.6-47.3]; *2*	**17.2** ± 7.8;<br>[5.9-31.3]; *0*	**23.4** ± 5.5;<br>[6.3–45.9]; *1*	**23.3** ± 4.8;<br>[0.0–52.5]; *0*	**31.6** ± 7.4;<br>[4.2–83.1]; *0*	**0.003**	0.021
Mϕ [%]:<br>**mean** ± 95% CI; [min–max]; *NA*	**2.7** ± 1.6;<br>[0.0–12.0]; *1*	**3.1** ± 2.3;<br>[0.0-12.0]; *1*	**2.1** ± 2.3;<br>[0.1-8.4]; *0*	**8.3** ± 5.6;<br>[0.0–39.7]; *1*	**4.4** ± 2.3;<br>[0.1–26.6]; *0*	**13.3** ± 4.4;<br>[0.0–46.6]; *0*	**<0.001**	0.178
MON-like cells [%]:<br>**mean** ± 95% CI; [min–max]; *NA*	**3.8** ± 1.9;<br>[0.1–16.6]; *1*	**2.5** ± 1.1;<br>[0.1-5.6]; *1*	**6.2** ± 5.6;<br>[0.4-16.6]; *0*	**13.0** ± 4.6;<br>[0.4–34.7]; *1*	**5.4** ± 1.9;<br>[0.3–19.4]; *0*	**14.7** ± 3.3;<br>[3.1–44.1]; *0*	**<0.001**	**0.002**
cDC2 [%]:<br>**mean** ± 95% CI; [min–max]; *NA*	**4.4** ± 2.9;<br>[0.2–23.6]; *1*	**4.7** ± 4.1;<br>[0.2-23.6]; *1*	**3.9** ± 4.2;<br>[0.3-15.3]; *0*	**11.8** ± 4.8;<br>[1.9–38.5]; *1*	**3.6** ± 3.3;<br>[0.1–45.3]; *0*	**21.7** ± 3.9;<br>[1.9–45.7]; *0*	**<0.001**	0.035

cDC1 [%]: **mean** ± 95% CI; [min–max]; *NA*	**3.5** ± 1.8; [0.1–16.9]; *1*	**4.0** ± 2.8; [0.1-16.9]; *1*	**2.7** ± 1.9; [0.1-7.3]; *0*	**5.5** ± 1.7; [0.4–15.0]; *0*	**1.8** ± 1.0; [0.1–8.9]; *0*	**5.3** ± 1.1; [1.2–15.2]; *0*	**<0.001**	0.056
cDC2/ cDC1 [ratio]: **mean** ± 95% CI; [min–max]; *NA*	**0.8** ± 0.5; [0.1-4.1]; *1*	**1.0** ± 0.8; [0.1-4.1]; *1*	**0.5** ± 0.3; [0.1-1.4]; *0*	**1.3** ± 0.9; [0.1-7.6]; *0*	**1.1** ± 0.5; [0.0-4.7]; *0*	**3.7** ± 0.8; [0.1-13.5]; *0*	**<0.001**	0.494
CD64$^+$ MON-Mϕ [MFI]: **mean** ± 95% CI; [min–max]; *NA*	**6,006** ± 703; [3,575–9,389]; *1*	**6,429** ± 852; [3,719-9,389]; *1*	**5,214** ± 1,314; [3,575-7,797]; *0*	**5,571** ± 997; [2,443–12,637]; *1*	**8,186** ± 1,148; [2,604–13,724]; *0*	**6,938** ± 1,947; [2,924–33,035]; *0*	**0.001**	0.108
cDC2 from MON-Mϕ [%]: **mean** ± 95% CI; [min–max]; *NA*	**14.4** ± 6.5; [1.1–58.9]; *1*	**13.9** ± 8.8; [1.1-58.9]; *1*	**15.3** ± 12.0; [2.0-42.9]; *0*	**15.8** ± 7.0; [2.0–54.7]; *0*	**11.5** ± 5.7; [0.4–63.9]; *0*	**36.1** ± 5.0; [0.5–67.5]; *0*	**<0.001**	0.816
cDC1 from MON -Mϕ [%]: **mean** ± 95% CI; [min–max]; *NA*	**25.7** ± 8.2; [3.2–77.3]; *1*	**26.3** ± 9.3; [3.2-58.3]; *1*	**24.6** ± 19.9; [6.6-77.3]; *0*	**22.1** ± 7.2; [0.6–60.9]; *0*	**15.2** ± 5.2; [1.2–41.5]; *0*	**12.2** ± 2.1; [3.5–28.8]; *0*	**0.007**	0.694
CD11b$^+$ MON -Mϕ [MFI]: **mean** ± 95% CI; [min–max]; *NA*	**10,157** ± 1,469; [4,629–16,206]; *1*	**9,801** ± 1,820; [4,629-15,848]; *1*	**10,825** ± 3,115; [4,983-16,206]; *0*	**8,303** ± 1,151; [3,941–12,426]; *1*	**13,921** ± 1,841; [7,247–24,886]; *0*	**9,414** ± 1,111; [5,155–18,976]; *0*	**<0.001**	0.153
CD64$^+$ CD11b$^+$ MON -Mϕ [%]: **mean** ± 95% CI; [min–max]; *NA*	**63.7** ± 11.1; [14.3–95.4]; *1*	**69.2** ± 12.7; [14.3-95.4]; *1*	**53.4** ± 23.9; [18.2-91.4]; *0*	**58.6** ± 10.6; [12.4–97.7]; *1*	**85.7** ± 5.6; [46.3–99.0]; *0*	**76.9** ± 4.7; [45.0–99.9]; *0*	**<0.001**	0.220
HLA-DR$^+$ CD11b$^+$ MON -Mϕ [MFI]: **mean** ± 95% CI; [min–max]; *NA*	**26,828** ± 7,393; [6,872–71,195]; *2*	**27,354** ± 11,215; [6,872-71,195]; *2*	**25,909** ± 9,604; [15,168-51,608]; *0*	**44,965** ± 16,801; [9,677–14,7359]; *1*	**33,385** ± 7,765; [5,796–86,183]; *0*	**51,258** ± 7,352; [11,400–12,3627]; *0*	**<0.001**	0.138
MON-like from CD11b$^+$ Mon-Mϕ [%]: **mean** ± 95% CI; [min–max]; *NA*	**36.3** ± 7.6; [8.7–67.1]; *1*	**31.9** ± 7.3; [8.7-55.7]; *1*	**44.7** ± 18.5; [13.5-67.1]; *0*	**37.1** ± 7.2; [4.8–59.3]; *1*	**43.0** ± 8.2; [14.9–93.1]; *0*	**29.1** ± 4.9; [9.2–71.0]; *0*	**0.028**	0.263
cDC2 from CD11b$^+$ Mon-Mϕ [%]: **mean** ± 95% CI; [min–max]; *NA*	**34.3** ± 11.6; [2.8–86.9]; *1*	**37.4** ± 15.9; [2.8-86.9]; *1*	**28.5** ± 19.5; [7.7-80.8]; *0*	**42.5** ± 11.2; [3.6–94.5]; *1*	**23.8** ± 8.4; [0.8–77.9]; *0*	**45.0** ± 6.2; [3.3–79.6]; *0*	**<0.001**	0.390
CD64$^+$ Mϕ [%]: **mean** ± 95% CI; [min–max]; *NA*	**81.0** ± 7.8; [34.5–100.0]; *1*	**85.3** ± 5.8; [60.7-100.0]; *1*	**72.8** ± 21.8; [34.5-97.2]; *0*	**86.3** ± 7.7; [38.1–100.0]; *1*	**94.9** ± 3.6; [53.4–100.0]; *0*	**95.8** ± 1.7; [76.6–100.0]; *0*	**<0.001**	0.351
TLT-2$^+$ Mϕ [%]: **mean** ± 95% CI; [min–max]; *NA*	**11.3** ± 8.7; [0.2–41.1]; *11*	**13.4** ± 12.9; [0.2-41.1]; *7*	**6.4** ± 8.9; [0.7-13.4]; *4*	**4.7** ± 2.5; [0.3–16.8]; *4*	**7.6** ± 3.0; [0.1–23.6]; *5*	**0.9** ± 0.5; [0.0–6.0]; *5*	**0.004**	0.740
IREM-2 Mϕ [MFI]: **mean** ± 95% CI; [min–max]; *NA*	**8,858** ± 3,193; [2,799–20,184]; *11*	**9,338** ± 4,803; [2,799-20,184]; *7*	**7,779** ± 3,661; [4,701-9,974]; *4*	**7,394** ± 1,582; [3,359–13,174]; *4*	**8,923** ± 3,676; [2,115–43,064]; *5*	**4,118** ± 875; [1,495–10,675]; *5*	**<0.001**	0.867
CD64$^+$ MON-like cells [%]: **mean** ± 95% CI; [min–max]; *NA*	**68.8** ± 11.5; [12.8–97.4]; *1*	**75.1** ± 12.2; [31.0-97.4]; *1*	**57.0** ± 26.3; [12.8-96.7]; *0*	**69.6** ± 11.6; [17.1–99.7]; *1*	**90.6** ± 5.6; [30.9–99.9]; *0*	**88.6** ± 3.5; [57.9–100.0]; *0*	**<0.001**	0.383
CD64$^+$ MON-like cells [MFI]: **mean** ± 95% CI; [min–max]; *NA*	**5,215** ± 525; [3,353–7,203]; *1*	**5,422** ± 579; [3,881-7,138]; *1*	**4,825** ± 1,236; [3,353-7,203]; *0*	**5,497** ± 1,002; [2,334–11,621]; *1*	**7,269** ± 901; [3,395–14,339]; *0*	**6,793** ± 1,027; [3,051–19,209]; *0*	**<0.001**	0.434
TLT-2$^+$ MON-like cells [%]: **mean** ± 95% CI; [min–max]; *NA*	**0.4** ± 0.4; [0.0–2.2]; *11*	**0.4** ± 0.6; [0.0-2.2]; *8*	**0.3** ± 0.4; [0.0-0.8]; *3*	**0.7** ± 0.9; [0.0–7.7]; *3*	**1.1** ± 0.7; [0.1–7.6]; *4*	**0.3** ± 0.1; [0.0–1.0]; *3*	**0.003**	0.998
CD88$^+$ MON-like cells [%]: **mean** ± 95% CI; [min–max]; *NA*	**89.2** ± 7.2; [63.0–99.6]; *11*	**90.1** ± 10.2; [63.0-99.6]; *8*	**87.7** ± 15.6; [73.7-97.1]; *3*	**86.1** ± 8.4; [45.7–99.9]; *3*	**92.3** ± 3.5; [65.5–100.0]; *4*	**93.9** ± 2.0; [78.1–100.0]; *3*	**0.016**	0.896
CD88$^+$ MON-like cells [MFI]: **mean** ± 95% CI; [min–max]; *NA*	**7,923** ± 2,609; [2,849–15,856]; *11*	**7,985** ± 3,306; [3,151-15,782]; *8*	**7,824** ± 6,630; [2,849-15,856]; *3*	**8,837** ± 3,413; [2,629–29,377]; *3*	**11,364** ± 1,894; [4,585–22,932]; *4*	**10,002** ± 1,246; [5,103–19,256]; *3*	**0.039**	0.937
IREM-2 MON-like cells [%]: **mean** ± 95% CI; [min–max]; *NA*	**55.6** ± 15.0; [2.5–92.8]; *11*	**58.0** ± 22.4; [2.5-92.8]; *8*	**51.7** ± 29.3; [25.7-84.4]; *3*	**53.0** ± 12.1; [19.5–90.2]; *3*	**60.5** ± 9.6; [15.1–99.9]; *4*	**70.0** ± 8.0; [14.1–97.2]; *3*	**0.002**	0.672
IREM-2 MON-like cells [MFI]: **mean** ± 95% CI; [min–max]; *NA*	**3,671** ± 830; [1,484–5,799]; *11*	**3,717** ± 1,215; [1,484-5,381]; *8*	**3,598** ± 1,733; [2,288-5,799]; *3*	**3,533** ± 765; [1,924–7,430]; *3*	**5,330** ± 2,133; [1,709–26,134]; *4*	**2,962** ± 490; [1,256–6,253]; *3*	**0.014**	0.832
CD64$^+$ cDC2 [%]: **mean** ± 95% CI; [min–max]; *NA*	**52.1** ± 11.6; [2.3–91.5]; *1*	**58.0** ± 13.4; [9.3-90.9]; *1*	**41.0** ± 24.9; [2.3-91.5]; *0*	**39.3** ± 10.9; [3.4–88.7]; *1*	**71.1** ± 6.8; [27.1–97.2]; *0*	**59.9** ± 8.0; [16.8–98.4]; *0*	**<0.001**	0.079
CD88$^+$ cDC1 [%]: **mean** ± 95% CI; [min–max]; *NA*	**57.1** ± 12.0; [8.3–91.9]; *8*	**60.7** ± 19.1; [8.3-91.9]; *6*	**51.1** ± 14.1; [30.2-66.7]; *2*	**44.8** ± 10.1; [22.4–83.8]; *4*	**38.7** ± 9.4; [8.0–72.2]; *10*	**48.3** ± 6.8; [7.6–89.3]; *3*	**0.040**	0.151
CD11b$^+$ NEU [MFI]: **mean** ± 95% CI; [min–max]; *NA*	**9,097** ± 1,175; [5,075–15,223]; *3*	**9,505** ± 1,896; [5,075-15,223]; *3*	**8,436** ± 970; [7,244-10,378]; *0*	**6,743** ± 730; [4,575–9,393]; *2*	**8,556** ± 901; [5,088–13,486]; *0*	**6,273** ± 601; [3,919–11,199]; *0*	**<0.001**	**0.004**
CD64$^+$ NEU [MFI]: **mean** ± 95% CI; [min–max]; *NA*	**689** ± 211; [162–2,221]; *3*	**717** ± 300; [171-2,221]; *3*	**643** ± 362; [162-1,516]; *0*	**576** ± 148; [162–1,018]; *2*	**671** ± 120; [261–1,685]; *0*	**402** ± 68; [175–1,284]; *0*	**<0.001**	0.704
CD88$^+$ NEU [%]: **mean** ± 95% CI; [min–max]; *NA*	**56.6** ± 8.7; [10.0–87.9]; *0*	**55.8** ± 11.9; [10.0-87.9]; *0*	**58.0** ± 15.2; [28.1-81.2]; *0*	**61.5** ± 13.9; [7.3–100.0]; *0*	**81.9** ± 5.5; [42.2–100.0]; *0*	**94.4** ± 3.6; [51.1–100.0]; *0*	**<0.001**	0.726
TLT-2$^+$ NEU [%]: **mean** ± 95% CI; [min–max]; *NA*	**4.4** ± 2.2; [0.0–16.6]; *0*	**4.7** ± 2.8; [0.0-15.7]; *0*	**3.9** ± 4.6; [0.1-16.6]; *0*	**2.8** ± 2.6; [0.0–25.0]; *0*	**2.4** ± 0.8; [0.1–8.7]; *0*	**0.3** ± 0.2; [0.0–2.8]; *0*	**<0.001**	0.367

TREM1$^+$ NEU [%]:<br>**mean** ± 95% CI; [min–max]; *NA*	**50.5** ± 7.6;<br>[13.1–85.7]; *0*	**52.2** ± 10.7;<br>[13.1-85.7]; *0*	**47.0** ± 10.9;<br>[25.4-71.6]; *0*	**40.7** ± 10.8;<br>[0.8–93.3]; *0*	**40.7** ± 6.7;<br>[21.0–78.7]; *0*	**32.0** ± 8.2;<br>[0.0–85.1]; *0*	**0.009**	0.180
CCR1$^+$ NEU [%]:<br>**mean** ± 95% CI; [min–max]; *NA*	**58.7** ± 7.4;<br>[42.7–76.4]; *11*	**61.5** ± 10.7;<br>[44.5-76.4]; *8*	**54.2** ± 13.4;<br>[42.7-70.5]; *3*	**45.8** ± 10.9;<br>[33.1–61.1]; *14*	**39.3** ± 9.8;<br>[17.0–67.2]; *15*	**60.0** ± 148.7;<br>[48.3–71.7]; *34*	**0.010**	0.080
CCR2$^+$ NEU [%]:<br>**mean** ± 95% CI; [min–max]; *NA*	**36.4** ± 10.7;<br>[11.3–64.9]; *11*	**43.0** ± 14.7;<br>[16.2-64.9]; *8*	**25.9** ± 16.1;<br>[11.3-44.2]; *3*	**8.0** ± 5.9;<br>[0.3–17.3]; *14*	**17.6** ± 8.2;<br>[0.6–38.1]; *15*	**2.4** ± 26.4;<br>[0.4–4.5]; *34*	**0.001**	**0.002**
CCR5$^+$ NEU [%]:<br>**mean** ± 95% CI; [min–max]; *NA*	**62.3** ± 10.8;<br>[32.5–85.3]; *11*	**66.8** ± 14.4;<br>[34.9-85.3]; *8*	**55.2** ± 22.8;<br>[32.5-81.8]; *3*	**73.1** ± 15.0;<br>[48.8–91.5]; *14*	**80.5** ± 13.8;<br>[37.1–100.0]; *15*	**93.7** ± 80.0;<br>[87.4–100.0]; *34*	**0.015**	0.249
NEU/ CCR7 [%]:<br>**mean** ± 95% CI; [min–max]; *NA*	**41.7** ± 6.4;<br>[23.5–59.1]; *11*	**43.1** ± 7.8;<br>[31.7-53.4]; *8*	**39.4** ± 16.2;<br>[23.5-59.1]; *3*	**24.5** ± 6.1;<br>[17.6–31.4]; *14*	**29.7** ± 9.4;<br>[0.2–53.0]; *15*	**39.8** ± 47.6;<br>[36.0–43.5]; *34*	**0.015**	**0.006**
NEU/ CXCR1 [%]:<br>**mean** ± 95% CI; [min–max]; *NA*	**91.2** ± 4.5;<br>[77.5-99.9]; *11*	**95.1** ± 3.5;<br>[88.7-99.9]; *8*	**85.0** ± 9.0;<br>[77.5-92.3]; *3*	**84.1** ± 19.3;<br>[55.7-99.0]; *14*	**78.3** ± 15.9;<br>[24.1-100.0]; *15*	**96.0** ± 50.2;<br>[92.1-100.0]; *34*	0.599	0.108
NEU/ CXCR1 [MFI]:<br>**mean** ± 95% CI; [min–max]; *NA*	**9,380** ± 1,663;<br>[5,416-13,892]; *11*	**10,712** ± 2,112;<br>[6,160-13,892]; *8*	**7,250** ± 1,899;<br>[5,416-9,620]; *3*	**6,028** ± 2,112;<br>[3,497-9,115]; *14*	**7,202** ± 2,308;<br>[2,236-14,686]; *15*	**6,767** ± 35,990;<br>[3,934-9,599]; *34*	0.109	**0.012**
CXCR2$^+$ NEU [%]:<br>**mean** ± 95% CI; [min–max]; *NA*	**74.1** ± 7.9;<br>[50.9–95.0]; *11*	**81.9** ± 5.6;<br>[73.6-95.0]; *8*	**61.6** ± 13.4;<br>[50.9-75.9]; *3*	**69.8** ± 16.3;<br>[55.8–95.7]; *14*	**87.2** ± 9.3;<br>[50.3–99.8]; *15*	**93.2** ± 59.1;<br>[88.5–97.8]; *34*	**0.013**	**0.021**
CXCR2$^+$ NEU [MFI]:<br>**mean** ± 95% CI; [min–max]; *NA*	**2,880** ± 433;<br>[1,863–4,099]; *11*	**3,225** ± 570;<br>[2,346-4,099]; *8*	**2,328** ± 409;<br>[1,863-2,782]; *3*	**2,031** ± 359;<br>[1,731–2,583]; *14*	**3,011** ± 924;<br>[1,689–7,529]; *15*	**2,000** ± 4,225;<br>[1,667–2,332]; *34*	**0.030**	**0.004**
NEU/ CXCR4 [%]:<br>**mean** ± 95% CI; [min–max]; *NA*	**70.8** ± 8.8;<br>[44.5-97.9]; *11*	**78.5** ± 9.3;<br>[62.9-97.9]; *8*	**58.4** ± 13.2;<br>[44.5-73.4]; *3*	**53.2** ± 18.7;<br>[25.6-76.6]; *14*	**49.9** ± 13.5;<br>[12.1-82.8]; *15*	**59.6** ± 186.8;<br>[44.9-74.3]; *34*	0.093	**0.012**
CCR7- CXCR4$^+$ NEU [%]:<br>**mean** ± 95% CI; [min–max]; *NA*	**15.1** ± 8.5;<br>[2.6-41.6]; *11*	**22.0** ± 11.8;<br>[5.3-41.6]; *8*	**4.1** ± 2.4;<br>[2.6-6.2]; *3*	**8.3** ± 9.4;<br>[0.1-22.9]; *14*	**5.9** ± 2.5;<br>[0.0-14.6]; *15*	**1.7** ± 19.3;<br>[0.2-3.2]; *34*	0.171	**0.024**
CCR7$^+$ CXCR4$^+$ NEU [%]:<br>**mean** ± 95% CI; [min–max]; *NA*	**33.6** ± 6.5;<br>[17.8–51.4]; *11*	**35.9** ± 8.0;<br>[23.1-51.4]; *8*	**29.8** ± 15.5;<br>[17.8-50.8]; *3*	**14.1** ± 7.7;<br>[2.5–24.2]; *14*	**17.5** ± 9.1;<br>[0.0–41.6]; *15*	**5.8** ± 58.1;<br>[1.2–10.4]; *34*	**0.006**	**0.003**
CCR7$^+$ CXCR4$^-$ NEU [%]:<br>**mean** ± 95% CI; [min–max]; *NA*	**5.3** ± 2.9;<br>[0.2-15.9]; *11*	**3.9** ± 3.9;<br>[0.2-13.6]; *8*	**7.5** ± 5.9;<br>[4.0-15.9]; *3*	**7.8** ± 6.3;<br>[1.6-17.8]; *14*	**9.6** ± 5.6;<br>[0.1-34.3]; *15*	**26.6** ± 99.7;<br>[18.8-34.5]; *34*	0.080	0.137
CCR7$^-$ CXCR4$^-$ NEU [%]:<br>**mean** ± 95% CI; [min–max]; *NA*	**46.0** ± 9.8;<br>[11.5–75.6]; *11*	**38.2** ± 11.1;<br>[11.5-53.5]; *8*	**58.6** ± 15.8;<br>[40.2-75.6]; *3*	**69.8** ± 9.2;<br>[62.8–86.0]; *14*	**66.9** ± 9.8;<br>[41.4–93.9]; *15*	**65.8** ± 21.6;<br>[64.1–67.5]; *34*	**0.005**	**0.001**
CXCR1$^-$ CCR2$^+$ NEU [%]:<br>**mean** ± 95% CI; [min–max]; *NA*	**0.0** ± 0.0;<br>[0.0-0.0]; *11*	**0.0** ± 0.0;<br>[0.0-0.0]; *8*	**0.0** ± 0.0;<br>[0.0-0.0]; *3*	**0.0** ± 0.0;<br>[0.0-0.0]; *14*	**0.0** ± 0.0;<br>[0.0-0.1]; *15*	**0.0** ± 0.1;<br>[0.0-0.0]; *34*	0.860	0.601
CXCR1$^+$ CCR2$^+$ NEU [%]:<br>**mean** ± 95% CI; [min–max]; *NA*	**36.1** ± 10.7;<br>[10.8–64.7]; *11*	**42.7** ± 14.7;<br>[15.9-64.7]; *8*	**25.5** ± 16.0;<br>[10.8-43.4]; *3*	**7.8** ± 5.8;<br>[0.3–16.9]; *14*	**17.3** ± 8.1;<br>[0.6–37.8]; *15*	**2.2** ± 24.1;<br>[0.3–4.1]; *34*	**0.001**	**0.002**
CXCR1$^+$ CCR2$^-$ NEU [%]:<br>**mean** ± 95% CI; [min–max]; *NA*	**53.7** ± 10.4;<br>[25.8–81.5]; *11*	**51.5** ± 16.3;<br>[25.8-81.5]; *8*	**57.3** ± 17.7;<br>[42.4-79.1]; *3*	**74.2** ± 18.3;<br>[51.5–93.7]; *14*	**59.0** ± 13.7;<br>[19.4–89.4]; *15*	**92.4** ± 43.2;<br>[89.0–95.8]; *34*	**0.041**	0.146
CXCR1$^-$ CCR2$^-$ NEU [%]:<br>**mean** ± 95% CI; [min–max]; *NA*	**10.2** ± 5.0;<br>[0.2-25.7]; *11*	**5.8** ± 4.0;<br>[0.2-12.4]; *8*	**17.2** ± 9.7;<br>[9.3-25.7]; *3*	**18.0** ± 20.7;<br>[1.5-48.2]; *14*	**23.6** ± 16.7;<br>[0.1-79.7]; *15*	**5.3** ± 67.1;<br>[0.0-10.6]; *34*	0.642	0.096
CXCR1$^+$ CD62L$^+$ NEU [%]:<br>**mean** ± 95% CI; [min–max]; *NA*	**8.8** ± 7.5;<br>[0.0–36.4]; *11*	**9.4** ± 11.2;<br>[0.0-36.4]; *8*	**7.9** ± 15.0;<br>[0.3-29.0]; *3*	**18.7** ± 16.4;<br>[0.9–41.3]; *14*	**26.4** ± 10.1;<br>[5.3–59.8]; *15*	**40.2** ± 416.9;<br>[7.4–73.0]; *34*	**0.024**	0.367
CXCR1$^+$ CD62L$^-$ NEU [%]:<br>**mean** ± 95% CI; [min–max]; *NA*	**81.9** ± 8.1;<br>[59.8–98.1]; *11*	**85.4** ± 11.6;<br>[59.8-98.1]; *8*	**76.2** ± 14.4;<br>[61.9-91.4]; *3*	**64.6** ± 20.7;<br>[43.4–95.7]; *14*	**51.2** ± 13.9;<br>[10.6–84.1]; *15*	**55.4** ± 360.2;<br>[27.0–83.7]; *34*	**0.009**	0.108
CXCR1$^-$ CD62L$^+$ NEU [%]:<br>**mean** ± 95% CI; [min–max]; *NA*	**0.5** ± 0.5;<br>[0.0-2.1]; *11*	**0.4** ± 0.6;<br>[0.0-1.7]; *8*	**0.7** ± 1.1;<br>[0.0-2.1]; *3*	**1.6** ± 2.4;<br>[0.0-5.9]; *14*	**5.6** ± 5.5;<br>[0.0-28.7]; *15*	**0.1** ± 1.4;<br>[0.0-0.2]; *34*	0.100	0.205
CXCR1$^-$ CD62L$^-$ NEU [%]:<br>**mean** ± 95% CI; [min–max]; *NA*	**8.8** ± 4.5;<br>[0.1-23.1]; *11*	**4.8** ± 3.7;<br>[0.1-11.7]; *8*	**15.1** ± 8.8;<br>[8.0-23.1]; *3*	**15.1** ± 19.0;<br>[1.1-45.6]; *14*	**16.9** ± 11.5;<br>[0.0-58.3]; *15*	**4.3** ± 54.9;<br>[0.0-8.7]; *34*	0.762	0.096
CXCR2$^+$ CXCR4$^+$ NEU [%]:<br>**mean** ± 95% CI; [min–max]; *NA*	**64.5** ± 8.5;<br>[39.4-86.4]; *11*	**72.1** ± 8.2;<br>[57.5-86.4]; *8*	**52.3** ± 13.9;<br>[39.4-69.2]; *3*	**45.2** ± 16.2;<br>[20.1-66.9]; *14*	**44.8** ± 13.5;<br>[9.0-77.7]; *15*	**48.7** ± 208.4;<br>[32.3-65.1]; *34*	0.052	**0.004**
CXCR2$^+$ CXCR4$^-$ NEU [%]:<br>**mean** ± 95% CI; [min–max]; *NA*	**15.7** ± 5.3;<br>[1.9–28.9]; *11*	**14.7** ± 8.0;<br>[1.9-28.9]; *8*	**17.3** ± 10.4;<br>[6.0-28.9]; *3*	**34.7** ± 23.3;<br>[20.4–78.1]; *14*	**46.7** ± 14.2;<br>[19.9–85.3]; *15*	**49.7** ± 194.4;<br>[34.4–65.0]; *34*	**<0.001**	0.056
CXCR2$^-$ CXCR4$^+$ NEU [%]:<br>**mean** ± 95% CI; [min–max]; *NA*	**3.7** ± 1.7;<br>[0.5–10.8]; *11*	**4.1** ± 2.7;<br>[0.5-10.8]; *8*	**3.1** ± 2.7;<br>[1.3-6.4]; *3*	**3.5** ± 4.0;<br>[0.0–8.2]; *14*	**0.9** ± 0.9;<br>[0.0–5.2]; *15*	**0.1** ± 0.4;<br>[0.1–0.2]; *34*	**0.008**	0.732
CXCR2$^-$ CXCR4$^-$ NEU [%]:<br>**mean** ± 95% CI; [min–max]; *NA*	**16.1** ± 7.3;<br>[0.9–38.7]; *11*	**9.1** ± 6.0;<br>[0.9-19.5]; *8*	**27.3** ± 11.6;<br>[14.5-38.7]; *3*	**16.6** ± 12.2;<br>[1.8–34.9]; *14*	**7.5** ± 6.5;<br>[0.1–32.2]; *15*	**1.5** ± 13.7;<br>[0.4–2.5]; *34*	**0.037**	**0.022**

α-defensins [μg/ml]:<br>**mean** ± 95% CI; [min–max]; *NA*	**4.9** ± 2.2;<br>[0.0–14.7]; *0*	**6.1** ± 3.0;<br>[0.0-14.7]; *0*	**2.4** ± 2.4;<br>[0.3-8.8]; *0*	**0.0** ± 0.0;<br>[0.0–0.1]; *0*	**3.8** ± 1.1;<br>[0.0–11.7]; *0*	**0.0** ± 0.0;<br>[0.0–0.2]; *0*	**<0.001**	**<0.001**
CRP [ug/ml]:<br>**mean** ± 95% CI; [min–max]; *NA*	**16.6** ± 8.7;<br>[0.0–81.6]; *0*	**21.2** ± 12.0;<br>[0.0-81.6]; *0*	**7.4** ± 10.9;<br>[0.0-37.0]; *0*	**3.0** ± 3.2;<br>[0.0–27.5]; *0*	**8.0** ± 3.4;<br>[0.0–33.0]; *0*	**0.9** ± 0.8;<br>[0.0–13.0]; *0*	**<0.001**	**0.024**
PTX3 [ng/ml]:<br>**mean** ± 95% CI; [min–max]; *NA*	**40.1** ± 23.2;<br>[0.0–162.2]; *0*	**56.8** ± 32.5;<br>[0.0-162.2]; *0*	**6.7** ± 7.2;<br>[0.0-25.0]; *0*	**3.3** ± 4.2;<br>[0.0–34.9]; *0*	**20.3** ± 14.8;<br>[0.0–185.1]; *0*	**0.0** ± 0.0;<br>[0.0–0.8]; *0*	**<0.001**	**0.001**
CXCL13 [ng/ml]:<br>**mean** ± 95% CI; [min–max]; *NA*	**1.5** ± 0.7;<br>[0.0–5.7]; *0*	**1.6** ± 0.9;<br>[0.0-5.7]; *0*	**1.3** ± 1.1;<br>[0.0-3.6]; *0*	**1.2** ± 1.4;<br>[0.0–12.4]; *0*	**1.4** ± 1.0;<br>[0.0–10.1]; *0*	**0.3** ± 0.3;<br>[0.0–6.0]; *0*	**<0.001**	0.147
sCD14 [ng/ml]:<br>**mean** ± 95% CI; [min–max]; *NA*	**1.7** ± 0.2;<br>[1.3–3.0]; *0*	**1.7** ± 0.1;<br>[1.5-2.2]; *0*	**1.8** ± 0.5;<br>[1.3-3.0]; *0*	**2.1** ± 0.6;<br>[1.3–6.7]; *0*	**1.5** ± 0.2;<br>[0.0–2.8]; *0*	**1.1** ± 0.2;<br>[0.0–2.3]; *0*	**<0.001**	0.439
sTREM1 [ng/ml]:<br>**mean** ± 95% CI; [min–max]; *NA*	**26.8** ± 3.3;<br>[3.6–37.4]; *0*	**26.6** ± 4.6;<br>[3.6-35.9]; *0*	**27.2** ± 5.5;<br>[17.6-37.4]; *0*	**25.9** ± 4.6;<br>[9.7–42.0]; *0*	**21.4** ± 3.9;<br>[6.4–39.9]; *0*	**12.0** ± 1.8;<br>[3.8–25.3]; *0*	**<0.001**	0.884

Legend: T-LYM: T-lymphocytes, MON–Mϕ: monocyte–macrophage lineage; NEUs: neutrophils; Mϕ: macrophage; MON-like: monocyte-like cells; cDC1: conventional dendritic cells type 1; cDC2: conventional dendritic cells type 2; $CD8^+$ T-LYM: cytotoxic T-lymphocytes; $CD4^+$ T-LYM: helper T-lymphocytes; CRP: C-reactive protein; PTX3: pentatrexin 3; PCT: procalcitonin.

Supplementary Figure S3. Receiver operating characteristic curves for synovial fluid parameters (α-defensins, total $CD45^+$ count, percentage of neutrophils, CRP and PTX3) between **(A)** both infection groups (PJI-all) and the osteolysis/aseptic loosening group (OL/AL) and **(B)** the microbiologically confirmed prosthetic joint infection group (PJI) and low-grade PJI (LG-PJI).

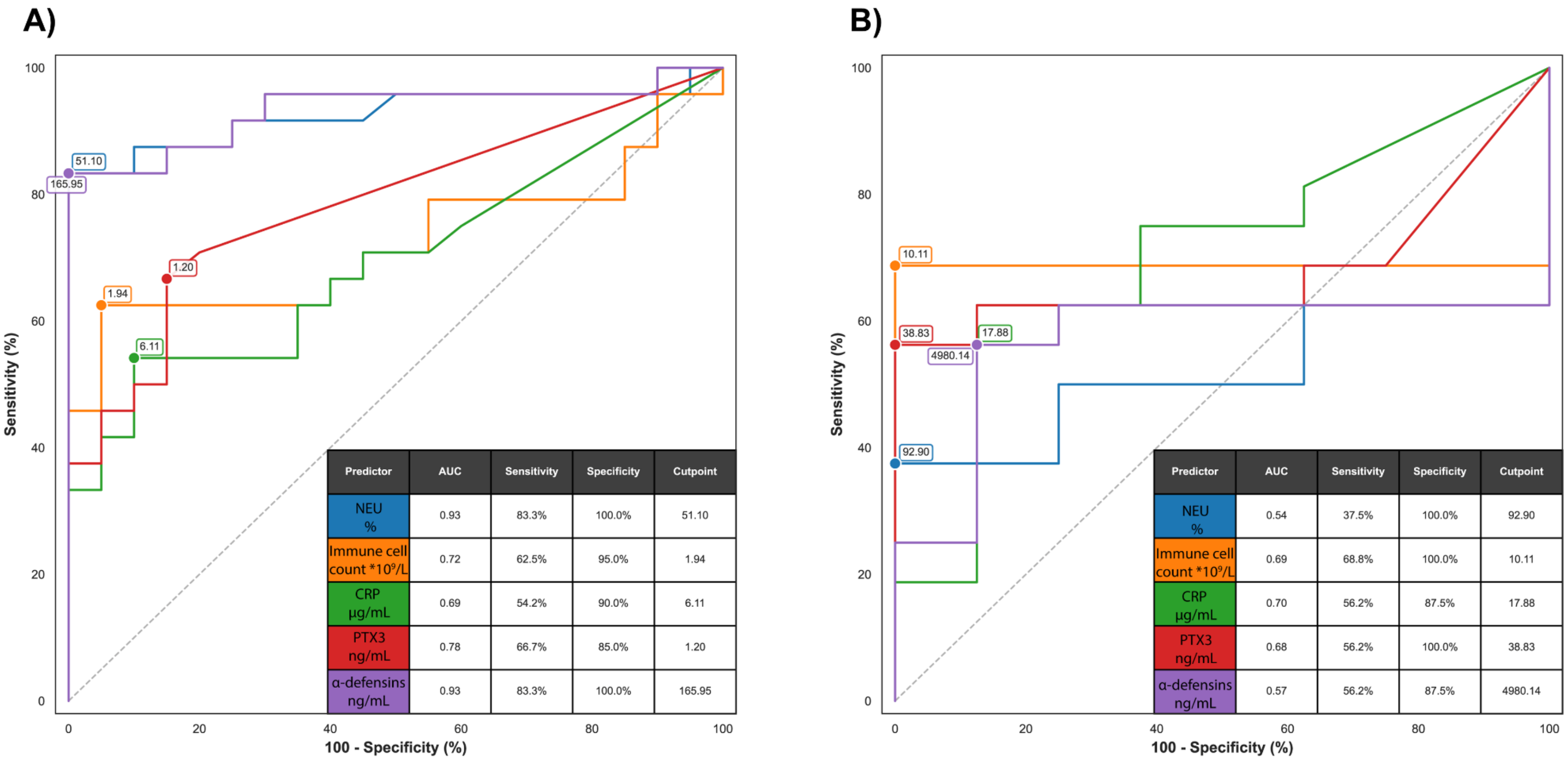

Supplementary Table S4. Distribution of synovial fluid-derived immune cell parameters and concentration of soluble mediators in synovial fluid in clusters of patients with total joint arthroplasty obtained using a patient similarity network.

	C1	C2	C3	C4
No. of patients	18	13	6	4
Sex (men/women)	9/9	12/1	4/2	3/1
Knee/hip	14/4	12/1	3/3	4/0
Age at SF sampling (years): **mean** ± 95% CI; [min–max]	**71.2** ± 5.9; [45.0–90.0]; *0*	**72.9** ± 3.5; [63.0–80.0]; *0*	**63.8** ± 10.6; [44.0–71.0]; *0*	**64.5** ± 16.5; [50.0–72.0]; *0*
Time since joint difficulties started in months: **mean** ± 95% CI; [min–max]; *NA*	**15.9** ± 10.7; [0.0–60.0]; *4*	**20.4** ± 15.0; [1.0–72.0]; *1*	**19.2** ± 17.7; [2.0–48.0]; *0*	**4.2** ± 6.8; [1.0–10.0]; *0*
Time since implant placement in months: **mean** ± 95% CI; [min–max]; *NA*	**52.2** ± 45.6; [1.0–252.0]; *2*	**51.3** ± 53.5; [1.0–276.0]; *0*	**5.2** ± 1.0; [4.0–6.0]; *1*	**10.2** ± 5.6; [5.0–12.0]; *0*
Cement: yes/no/hybrid /*NA*	12/3/0/*3*	13/0/0/*0*	2/2/1/*1*	4/0/0/*0*
Cement type: Biomet Optipac/Copal G+C/ Refobacin/none/*NA*	4/0/2/3/*9*	4/1/1/0/*7*	1/0/0/2/*1*	0/0/1/0/*3*
BMI [kg/m^2]: **mean** ± 95% CI; [min–max]; *NA*	**31.4** ± 2.2; [24.1–40.9]; *1*	**29.9** ± 2.7; [24.0–39.5]; *0*	**29.4** ± 2.8; [26.2–32.5]; *1*	**36.1** ± 11.8; [29.6–45.9]; *0*
Pain level in VAS[a]: **mean** ± 95% CI; [min–max]	**4.0** ± 0.8; [0.0–7.0]; *0*	**5.1** ± 0.9; [3.0–8.0]; *0*	**3.7** ± 0.5; [3.0–4.0]; *0*	**4.5** ± 0.9; [4.0–5.0]; *0*
Pain level[b]: 0/1/2/3/*NA*	1/1/14/2/*0*	0/0/10/3/*0*	0/1/3/2/*0*	0/0/3/1/*0*
Fluid volume (ml): **mean** ± 95% CI; [min–max]	**19.8** ± 7.6; [3.0–50.0]; *0*	**24.2** ± 12.6; [2.5–65.0]; *0*	**22.8** ± 24.1; [3.5–64.0]; *0*	**14.5** ± 17.5; [4.0–30.0]; *0*
Viscosity: low/normal/high	11/5/2	8/4/1	2/4/0	0/4/0
Colour: transparent/yellow/cloudy/orange/*NA*	0/1/10/7/*0*	0/0/9/4/*0*	0/0/4/2/*0*	0/0/3/1/*0*
Total CD45$^+$ count [×10^9/l]; **mean** ± 95% CI; [min–max]	**0.5** ± 0.2; [0.1–1.7]; *0*	**17.1** ± 15.8; [0.4–88.6]; *0*	**3.4** ± 3.8; [0.6–9.7]; *0*	**76.9** ± 106.1; [18.7–149.1]; *0*
Percentage of CD45$^+$ cells [%]: **mean** ± 95% CI; [min–max]	**8.1** ± 4.9; [0.1–32.8]; *0*	**35.6** ± 18.9; [0.1–86.9]; *0*	**28.3** ± 31.0; [1.2–83.1]; *0*	**54.4** ± 38.9; [30.4–75.8]; *0*
LYM [%]: **mean** ± 95% CI; [min–max]	**51.4** ± 11.2; [13.4–89.1]; *0*	**6.5** ± 4.0; [0.8–23.6]; *0*	**23.9** ± 18.2; [4.8–52.1]; *0*	**3.2** ± 6.6; [0.7–9.4]; *0*
MON–Mϕ [%]: **mean** ± 95% CI; [min–max]	**27.1** ± 10.0; [6.0–69.3]; *0*	**11.1** ± 6.3; [1.0–28.8]; *0*	**37.6** ± 28.9; [6.8–77.9]; *0*	**8.1** ± 12.3; [0.5–17.3]; *0*
NEU [%]: **mean** ± 95% CI; [min–max]	**20.5** ± 9.0; [2.5–65.9]; *0*	**81.4** ± 9.3; [47.0–97.9]; *0*	**37.2** ± 30.0; [10.2–87.9]; *0*	**87.9** ± 11.9; [80.3–98.1]; *0*
EOS-like cells [%]: **mean** ± 95% CI; [min–max]	**2.3** ± 2.3; [0.0–15.9]; *0*	**4.5** ± 3.6; [0.6–22.9]; *0*	**0.4** ± 0.2; [0.0–0.6]; *0*	**4.7** ± 10.3; [0.3–14.3]; *0*
CD4$^+$/CD8$^+$ T-LYM [ratio]: **mean** ± 95% CI; [min–max]	**1.6** ± 0.6; [0.6–5.1]; *0*	**1.9** ± 0.4; [0.6–3.0]; *0*	**2.0** ± 2.1; [0.6–5.9]; *0*	**5.1** ± 4.1; [3.1–8.8]; *0*
TLT-2$^+$ CD45RO$^+$ CD4$^+$ [%]: **mean** ± 95% CI; [min–max]; *NA*	**1.9** ± 0.8; [0.2–5.3]; *0*	**5.6** ± 3.0; [0.2–15.1]; *0*	**2.4** ± 2.3; [0.6–5.4]; *1*	**2.9** ± 1.9; [1.5–4.5]; *0*
TLT-2$^+$ CD8$^+$ T-LYM [%]: **mean** ± 95% CI; [min–max]; *NA*	**5.8** ± 2.0; [0.3–12.8]; *0*	**10.3** ± 5.8; [0.5–30.8]; *0*	**3.6** ± 4.4; [0.6–9.0]; *1*	**5.2** ± 4.6; [2.1–9.0]; *0*
NK cells from LYM [%]: **mean** ± 95% CI; [min–max]	**8.1** ± 3.4; [1.6–23.3]; *0*	**14.3** ± 3.1; [2.5–19.8]; *0*	**5.9** ± 2.6; [3.2–9.8]; *0*	**37.1** ± 5.7; [33.4–41.4]; *0*
HLA-DR$^+$ NK [%]: **mean** ± 95% CI; [min–max]	**24.7** ± 6.6; [6.3–47.3]; *0*	**11.9** ± 4.2; [3.6–27.5]; *0*	**22.8** ± 7.4; [14.1–32.6]; *0*	**6.0** ± 6.3; [0.6–10.1]; *0*
Mϕ [%]: **mean** ± 95% CI; [min–max]	**4.4** ± 3.7; [0.0–30.9]; *0*	**3.8** ± 2.6; [0.1–12.0]; *0*	**13.1** ± 17.7; [1.0–39.7]; *0*	**3.4** ± 8.1; [0.0–11.0]; *0*
MON-like cells [%]: **mean** ± 95% CI; [min–max]	**8.4** ± 3.9; [0.4–26.2]; *0*	**5.0** ± 3.6; [0.4–16.6]; *0*	**17.7** ± 11.3; [4.4–34.7]; *0*	**2.7** ± 3.6; [0.2–5.2]; *0*

cDC2 [%]:<br>**mean** ± 95% CI; [min–max]	**14.1** ± 5.1;<br>[1.9–38.5]; *0*	**2.0** ± 1.5;<br>[0.3–8.1]; *0*	**6.4** ± 4.8;<br>[0.5–10.7]; *0*	**1.7** ± 3.8;<br>[0.2–5.3]; *0*
HLA-DR$^+$ CD11b$^+$ MON-Mϕ [%]:<br>**mean** ±95% CI; [min–max]	**50,880** ± 17,037;<br>[14,267–14,7359]; *0*	**25,569** ± 8,399;<br>[6,872–51,608]; *0*	**22,609** ± 9,988;<br>[9,677–3,3331]; *0*	**15,169** ± 11,325;<br>[9,035–2,4261]; *0*
Mϕ from CD11b$^+$ MON-Mϕ [%]:<br>**mean** ± 95% CI; [min–max]	**12.9** ± 6.5;<br>[0.4–51.0]; *0*	**33.6** ± 13.7;<br>[4.2–69.9]; *0*	**26.1** ± 20.5;<br>[3.4–51.0]; *0*	**28.8** ± 39.6;<br>[7.0–63.4]; *0*
Mon-like cells from CD11b$^+$ MON-Mϕ [%]: **mean** ± 95% CI; [min–max]	**28.3** ± 7.1;<br>[4.8–58.5]; *0*	**41.5** ± 10.1;<br>[13.5–67.1]; *0*	**52.3** ± 10.1;<br>[39.1–65.8]; *0*	**39.1** ± 13.1;<br>[29.9–47.1]; *0*
cDC2 from CD11b$^+$ MON-Mϕ [%]:<br>**mean** ± 95% CI; [min–max]	**57.3** ± 10.8;<br>[15.3–94.5]; *0*	**19.7** ± 6.4;<br>[2.8–33.4]; *0*	**20.5** ± 16.2;<br>[3.6–41.9]; *0*	**28.1** ± 30.9;<br>[3.1–46.2]; *0*
CD88$^+$ MON-like cells [%]:<br>**mean** ± 95% CI; [min–max]; *NA*	**89.8** ± 7.6;<br>[66.7–99.9]; *5*	**90.0** ± 8.6;<br>[73.7–99.6]; *5*	**82.5** ± 23.7;<br>[45.7–98.2]; *0*	**73.7** ± 135.3;<br>[63.0–84.3]; *2*
TLT-2$^+$ Mϕ [%]:<br>**mean** ± 95% CI; [min–max]; *NA*	**4.8** ± 3.1;<br>[0.2–16.8]; *5*	**16.7** ± 13.6;<br>[0.7–41.1]; *5*	**3.3** ± 3.1;<br>[0.3–8.3]; *0*	**3.0** ± 8.4;<br>[2.3–3.6]; *2*
IREM-2 Mϕ [MFI]:<br>**mean** ± 95% CI; [min–max]; *NA*	**6,420** ± 1,505;<br>[3,359–10,179]; *5*	**11,332** ± 4,906;<br>[2,799–20,184]; *5*	**8,035** ± 2,690;<br>[6,173–12,640]; *0*	**5568** ± 9746;<br>[4801–6335]; *2*
CD88$^+$ cDC1 [%]:<br>**mean** ± 95% CI; [min–max]	**56.9** ± 9.2;<br>[23.4–83.8]; *3*	**54.7** ± 19.2;<br>[8.3–91.9]; *3*	**24.4** ± 2.6;<br>[22.4–26.4]; *2*	**30.1** ± nan;<br>[30.1–30.1]; *3*
CD11b$^+$ NEUs [MFI]:<br>**mean** ± 95% CI; [min–max]; *NA*	**7,229** ± 784.7;<br>[5,123–9,530]; *2*	**8,163** ± 1,292;<br>[4,575–12,913]; *0*	**6,754** ± 1,193;<br>[4,966–8,197]; *0*	**12,528** ± 4,905;<br>[8,440–15,223]; *0*
CD88$^+$ NEUs [%]:<br>**mean** ± 95% CI; [min–max]	**63.1** ± 13.8;<br>[10.0–100.0]; *0*	**57.6** ± 11.7;<br>[24.1–85.2]; *0*	**57.8** ± 39.5;<br>[7.3–91.9]; *0*	**47.9** ± 31.6;<br>[27.9–68.1]; *0*
TREM1$^+$ NEUs [%]:<br>**mean** ± 95% CI; [min–max]	**48.5** ± 10.4;<br>[10.4–79.3]; *0*	**45.4** ± 11.2;<br>[13.1–85.7]; *0*	**42.2** ± 27.3;<br>[25.4–93.3]; *0*	**53.8** ± 31.2;<br>[25.8–71.6]; *0*
TLT-2$^+$ NEUs [%]:<br>**mean** ± 95% CI; [min–max]	**3.4** ± 3.3;<br>[0.0–25.0]; *0*	**3.7** ± 2.6;<br>[0.1–13.6]; *0*	**1.7** ± 1.9;<br>[0.2–4.7]; *0*	**7.0** ± 9.4;<br>[2.8–15.7]; *0*
α-defensins [μg/ml]:<br>**mean** ± 95% CI; [min–max]	**0.1** ± 0.2;<br>[0.0–1.7]; *0*	**5.4** ± 3.0;<br>[0.1–13.9]; *0*	**0.8** ± 1.9;<br>[0.0–4.5]; *0*	**9.1** ± 10.8;<br>[0.3–14.7]; *0*
CRP [μg/ml]:<br>**mean** ± 95% CI; [min–max]	**2.3** ± 3.2;<br>[0.0–27.5]; *0*	**13.5** ± 9.9;<br>[0.0–40.1]; *0*	**5.7** ± 8.1;<br>[0.0–16.1]; *0*	**41.7** ± 43.5;<br>[20.7–81.6]; *0*
PTX3 [ng/ml]:<br>**mean** ± 95% CI; [min–max]	**2.2** ± 2.7;<br>[0.0–20.6]; *0*	**31.9** ± 27.5;<br>[0.0–129.7]; *0*	**7.9** ± 14.8;<br>[0.0–34.9]; *0*	**91.1** ± 94.6;<br>[40.1–162.2]; *0*
CXCL13 [ng/ml]:<br>**mean** ± 95% CI; [min–max]	**1.3** ± 1.5;<br>[0.0–12.4]; *0*	**1.6** ± 1.1;<br>[0.0–5.7]; *0*	**0.6** ± 0.8;<br>[0.0–2.0]; *0*	**2.5** ± 2.3;<br>[0.6–3.7]; *0*
sCD14 [ng/ml]:<br>**mean** ± 95% CI; [min–max]	**1.9** ± 0.4;<br>[1.3–3.8]; *0*	**1.7** ± 0.3;<br>[1.5–3.0]; *0*	**2.4** ± 2.2;<br>[1.3–6.7]; *0*	**2.0** ± 0.3;<br>[1.7–2.2]; *0*
sTREM1 [ng/ml]:<br>**mean** ± 95% CI; [min–max]	**26.6** ± 4.6;<br>[9.8–39.5]; *0*	**25.3** ± 3.8;<br>[12.1–32.0]; *0*	**26.8** ± 11.4;<br>[9.7–42.0]; *0*	**32.9** ± 4.9;<br>[29.0–35.9]; *0*
PCT [pg/ml]:<br>**mean** ± 95% CI; [min–max]	**97.3** ± 202.6;<br>[0.0–1729.4]; *0*	**103.7** ± 179.5;<br>[0.0–1054.5]; *0*	**85.9** ± 144.0;<br>[0.0–310.3]; *0*	**103.8** ± 330.3;<br>[0.0–415.1]; *0*

Legend: T-LYM: T-lymphocytes, MON–Mϕ: monocyte–macrophage lineage; NEUs: neutrophils; Mϕ: macrophage; MON-like: monocyte-like cells; cDC1: conventional dendritic cells type 1; cDC2: conventional dendritic cells type 2; CD8$^+$ T-LYM: cytotoxic T-lymphocytes; CD4$^+$ T-LYM: helper T-lymphocytes.

[a] Categorisation of pain level based on the self-reported 11-point visual analogue scale (VAS): 0 = no pain (VAS 0–1); 1 = mild pain (VAS 2–3); 2 = moderate pain (VAS 4–5); 3 = severe pain (VAS ≥ 6)

[b] Pain level according to self-reported 11-point VAS. 0 = VAS 0–1; 1 = VAS 2–3; 2 = VAS 4–5; 3 = VAS ≥ 6

Supplementary Table S5. Differences in immune cell patterns in synovial fluid between clusters of patients with total joint arthroplasty obtained through a patient similarity network: P-values from Mann–Whitney U test. Marked parameters have been used for network construction. Significant P-values are marked in bold.

	Mann-Whitney U-test P-value					
	**C1 vs C2**	**C1 vs C3**	**C1 vs C4**	**C2 vs C3**	**C2 vs C4**	**C3 vs C4**
No. of patients	18 vs 13	18 vs 6	18 vs 4	13 vs 6	13 vs 4	6 vs 4
Sex (men/women)	9/9 vs 12/1	9/9 vs 4/2	9/9 vs 3/1	12/1 vs 4/2	12/1 vs 3/1	4/2 vs 3/1
Knee/ hip	14/4 vs 12/1	14/4 vs 3/3	14/4 vs 4/0	12/1 vs 3/3	12/1 vs 4/0	3/3 vs 4/0
Time since joint difficulties started [months]	0.469	0.453	0.391	0.813	0.111	0.085
Time since implant placement [months]	0.217	0.835	0.567	0.052	0.557	0.098
BMI [kg/m^2]	0.263	0.359	0.275	1.000	0.130	0.111
Total CD45$^+$ count [×10^9/l]	**<0.001**	**0.002**	**<0.001**	0.368	**0.023**	**0.010**
LYM [%]	**0.000**	**0.015**	**<0.001**	**0.011**	0.202	**0.038**
MON-Mϕ [%]	**0.010**	0.378	**0.033**	**0.017**	0.703	**0.038**
NEUs [%]	**<0.001**	0.119	**<0.001**	**0.005**	0.412	0.067
Percentage of CD45$^+$ cells [%]	**0.038**	0.047	**0.001**	0.701	0.202	0.171
EOS-like cells [%]	**0.008**	0.689	0.141	**<0.001**	0.785	0.067
CD4$^+$/CD8$^+$ T-LYM [ratio]	0.093	0.974	**0.003**	0.416	**0.001**	0.067
TLT-2$^+$ CD45RO$^+$ CD4$^+$ T-LYM [%]	**0.021**	0.479	0.160	0.246	0.703	0.413
TLT-2$^+$ CD8$^+$ T-LYM [%]	0.242	0.403	0.902	0.143	0.412	0.556
NK from LYM [%]	**0.016**	0.974	**<0.001**	**0.005**	**0.001**	**0.010**
HLA-DR$^+$ NK [%]	**0.004**	0.739	**0.007**	**0.009**	0.202	**0.010**
Mϕ [%]	0.984	0.251	0.652	0.323	0.785	0.352
MON-like cells [%]	0.089	0.056	0.141	**0.007**	0.703	**0.019**
cDC2 [%]	**0.000**	0.066	**0.002**	**0.022**	0.956	0.171
HLA-DR$^+$ CD11b$^+$ MON-Mϕ [MFI]	**0.014**	**0.015**	**0.007**	0.831	0.245	0.257
Mϕ from CD11b$^+$ MON-Mϕ [%]	**0.006**	0.125	0.118	0.467	0.785	0.762
MON-like cells from CD11b$^+$ MON-Mϕ [%]	**0.025**	**<0.001**	0.173	0.244	0.785	0.114
cDC2 from CD11b$^+$ MON-Mϕ [%]	**<0.001**	**0.002**	**0.026**	0.966	0.412	0.610
CD88$^+$ MON-like cells	0.690	0.404	0.106	1.000	0.178	0.643
TLT-2$^+$ Mϕ [%]	0.076	0.792	1.000	0.108	0.533	1.000
IREM-2$^+$ Mϕ [%]	0.045	0.282	0.933	0.282	0.400	0.429
CD88$^+$ cDC1 [%]	0.849	**0.004**	0.250	0.024	0.364	0.400
CD11b$^+$ NEUs [MFI]	0.203	0.541	**0.005**	0.072	**0.015**	**0.010**
CD88$^+$ NEUs [%]	0.441	0.820	0.268	0.639	0.549	0.476
TREM1$^+$ NEUs [%]	0.650	0.310	0.733	0.368	0.350	0.476
TLT-2$^+$ NEUs [%]	0.123	0.617	**0.045**	0.510	0.102	0.069
CRP [μg/ml]	0.106	0.431	**0.003**	0.449	0.099	**0.014**
α-defensins [μg/ml]	**<0.001**	0.117	**0.003**	**0.005**	0.412	**0.019**
PTX3 [ng/ml]	**0.005**	0.198	**<0.001**	0.280	**0.035**	**0.013**
CXCL13 [pg/ml]	0.071	0.382	**0.040**	0.334	0.296	0.067
sCD14 [ng/ml]	0.395	0.790	0.262	0.639	**0.032**	0.257
sTREM1 [ng/ml]	0.622	1.000	0.300	0.701	**0.015**	0.257
PCT [pg/ml]	0.757	0.238	0.521	0.499	0.734	1.000

Legend: T-LYM: T-lymphocytes, MON–Mϕ: monocyte–macrophage lineage; NEUs: neutrophils; Mϕ: macrophage; MON-like: monocyte-like cells; cDC1: conventional dendritic cells type 1; cDC2: conventional dendritic cells type 2; CD8$^+$ T-LYM: cytotoxic T-lymphocytes; CD4$^+$ T-LYM: helper T-lymphocytes.

Table S6. The threshold values obtained from the ROC curve for individual parameters and their combination used for patient similarity networks construction.

PJI-all vs OL/AL				
**Parameter**	**Cut-off**	**AUC**	**Sensitivity (%)**	**Specificity (%)**
LYM [%]	15.3	0.88	95.0	79.2
NK cells from LYM [%]	12.2	0.79	69.6	84.2
HLA-DR$^+$ NK cells [%]	13.4	0.74	78.9	63.6
HLA-DR$^+$ CD11b$^+$ MON-Mϕ [MFI]	27501	0.68	73.7	68.2
cDC2 from CD11b$^+$ MON-Mϕ [%]	33.4	0.61	63.2	65.2
CD88$^+$ cDC1 [%]	56.9	0.69	56.2	81.2

PJI-all vs OL/AL				
**Parameters (multivariable RIDGE logistic regression mode)**	**Cut-off e (Youden's J statistic)**	**AUC (95% CI) (DeLong's method)**	**Sensitivity (%)**	**Specificity (%)**
LYM [%] + NK cells from LYM [%] + HLA-DR$^+$ NK cells [%] + HLA-DR$^+$ CD11b$^+$ MON-Mϕ [MFI] + cDC2 from CD11b$^+$ MON-Mϕ [%] + CD88$^+$ cDC1 [%]	0.69	0.99 (0.63-1.00)	86.7	100.0

Legend: T-LYM: T-lymphocytes, MON–Mϕ: monocyte–macrophage lineage; NEUs: neutrophils; Mϕ: macrophage; MON-like: monocyte-like cells; cDC1: conventional dendritic cells type 1; cDC2: conventional dendritic cells type 2; CD8$^+$ T-LYM: cytotoxic T-lymphocytes; CD4$^+$ T-LYM: helper T-lymphocytes; PJI-all: patients with microbiologically confirmed prosthetic joint infection and with low-grade PJI; PJI: patients with microbiologically confirmed prosthetic joint infection; OL/AL: patients with osteolysis or aseptic loosening of the implant; AUC: area under the curve.

Supplementary Figure S4. Receiver operating characteristic curves for individual and combined synovial fluid parameters used for patient similarity network construction **(A)** Separate parameters of LYM (%), NK cells from LYM (%), HLA-DR^{+} NK cells (%), cDC2 cells from CD11b^{+} MON-Mϕ (%), HLA-DR^{+} CD11b^{+} MON–Mϕ cells (MFI), CD88^{+} cDC1 (%); **(B)** Combination of these parameters.

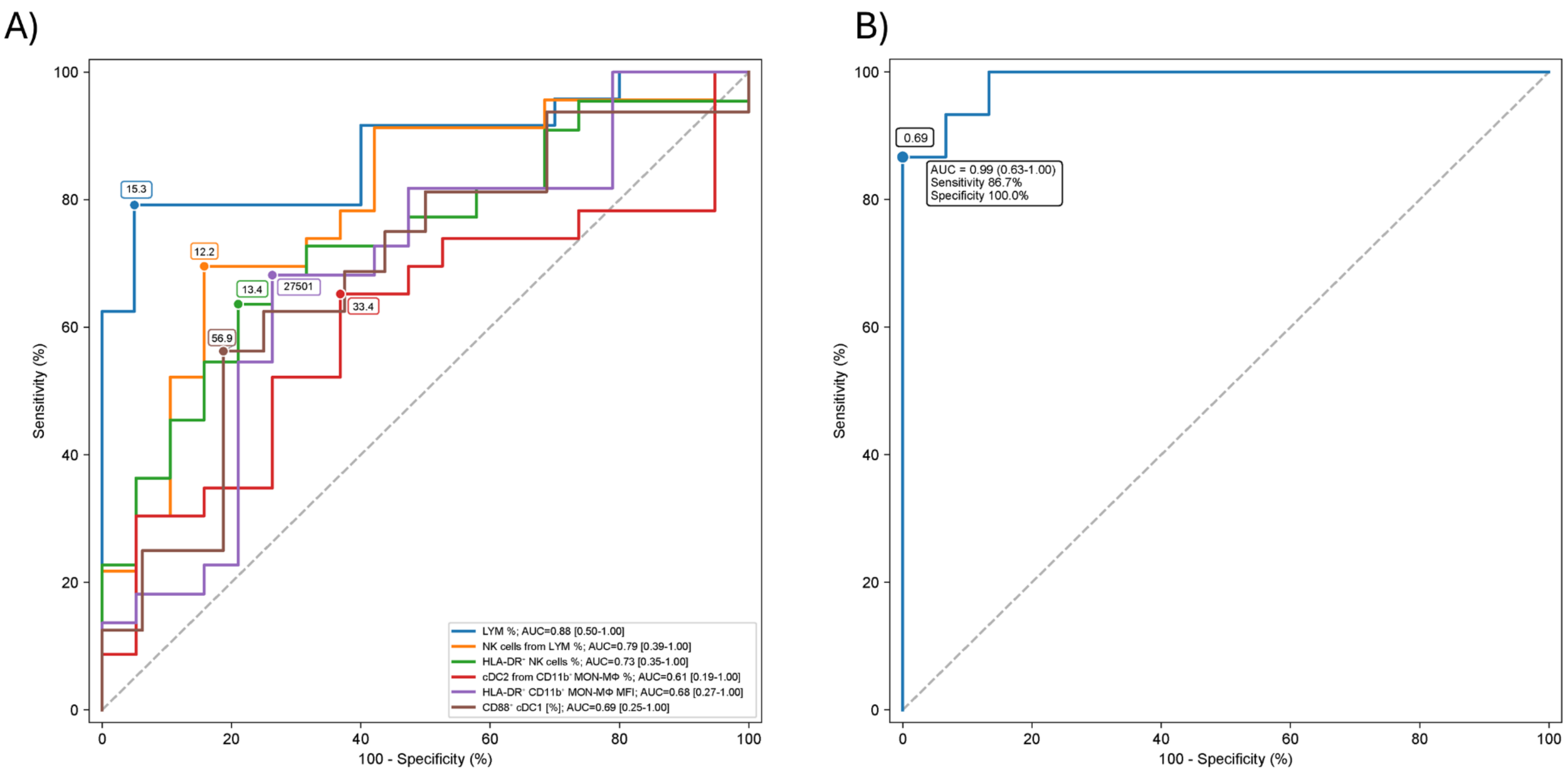

Legend: T-LYM: T-lymphocytes, MON–Mϕ: monocyte–macrophage lineage; NEUs: neutrophils; Mϕ: macrophage; MON-like: monocyte-like cells; cDC1: conventional dendritic cells type 1; cDC2: conventional dendritic cells type 2; CD8^{+} T-LYM: cytotoxic T-lymphocytes; CD4^{+} T-LYM: helper T-lymphocytes; PJI-all: patients with microbiologically confirmed prosthetic joint infection and with low-grade PJI; PJI: patients with microbiologically confirmed prosthetic joint infection; OL/AL: patients with osteolysis or aseptic loosening of the implant; AUC: area under the curve.

Supplementary Table S7. Differential expression of selected genes (expressed as transcript per million, TPM) in fibroblast-like synoviocytes and chondrocytes derived from patients with osteoarthritis cultured with/without Ti_6Al_4V alloy particles. Data from RNAseq are presented as the mean of three biological replicates.

Gene	ENSEMBL_ID	Fibroblasts-like synoviocytes			Chondrocytes		
		**Not stimulated TPM mean**	+ *Ti_6Al_4V* alloy *TPM mean*	**Fold change**	**Not stimulated TPM mean**	+ *Ti_6Al_4V* alloy *TPM mean*	**Fold change**
ADM	ENSG00000148926	66.5	117.2	1.8	45.3	43.6	1.0
AHR	ENSG00000106546	39.4	50.4	1.3	*nd*	*nd*	*nd*
ALPL	ENSG00000162551	14.9	14.3	1.0	*nd*	*nd*	*nd*
CCL2	ENSG00000108691	126.2	509.8	4.0	289.7	570.6	2.0
CCL5	ENSG00000271503	1.5	9.6	6.3	0.5	4.3	8.6
CCL7	ENSG00000108688	0.6	1.8	3.0	2.5	6.2	2.5
CSF2	ENSG00000164400	1.0	2.3	2.3	*nd*	*nd*	*nd*
CTSB	ENSG00000164733	*nd*	*nd*	*nd*	1033.1	1100.7	1.1
CTSL	ENSG00000135047	*nd*	*nd*	*nd*	680.9	777.5	1.1
CXCL1	ENSG00000163739	107.3	614.0	5.7	24.2	135.1	5.6
CXCL12	ENSG00000107562	*nd*	*nd*	*nd*	125.0	86.9	0.7
CXCL3	ENSG00000163734	11.9	91.5	7.7	8.9	44.4	5.0
CXCL5	ENSG00000163735	13.4	67.6	5.1	3.7	11.7	3.2
CXCL6	ENSG00000124875	158.6	1329.4	8.4	30.5	327.0	10.7
CXCL8	ENSG00000169429	8.9	255.6	28.7	13.1	173.6	13.3
FTH1	ENSG00000167996	6955.8	8961.7	1.3	6574.3	8102.4	1.2
GADD45A	ENSG00000116717	57.4	70.7	1.2	*nd*	*nd*	*nd*
GCH1	ENSG00000131979	1.8	3.8	2.1	*nd*	*nd*	*nd*
H1-2	ENSG00000187837	41.9	49.1	1.2	*nd*	*nd*	*nd*
IL1B	ENSG00000125538	*nd*	*nd*	*nd*	0.7	2.8	3.9
IL32	ENSG00000008517	29.8	67.5	2.3	27.5	44.8	1.6
IL33	ENSG00000137033	204.1	461.8	2.3	*nd*	*nd*	*nd*
IL6	ENSG00000136244	35.7	189.9	5.3	117.7	306.6	2.6
IRF7	ENSG00000185507	*nd*	*nd*	*nd*	6.7	9.4	1.4
ISG20	ENSG00000172183	*nd*	*nd*	*nd*	5.3	9.1	1.7
JUP	ENSG00000173801	1.1	0.7	0.6	*nd*	*nd*	*nd*
KCNJ2	ENSG00000123700	4.6	7.6	1.7	*nd*	*nd*	*nd*

LGALS3	ENSG00000131981	*nd*	*nd*	*nd*	238.3	269.7	1.1
MMP9	ENSG00000100985	1.3	6.9	5.3	0.6	1.5	2.4
MX1	ENSG00000157601	*nd*	*nd*	*nd*	1.2	1.8	1.5
NFKB1	ENSG00000109320	18.0	27.6	1.5	*nd*	*nd*	*nd*
OAS1	ENSG00000089127	*nd*	*nd*	*nd*	1.9	2.9	1.6
OAS1	ENSG00000089127	6.9	8.9	1.3	*nd*	*nd*	*nd*
PDE4B	ENSG00000184588	10.7	17.2	1.6	*nd*	*nd*	*nd*
PLAUR	ENSG00000011422	173.6	161.2	0.9	*nd*	*nd*	*nd*
PSMB9	ENSG00000240065	*nd*	*nd*	*nd*	16.2	26.0	1.6
PSMB9	ENSG00000240065	15.3	22.3	1.5	*nd*	*nd*	*nd*
PTGS2	ENSG00000073756	10.4	64.0	6.1	*nd*	*nd*	*nd*
PTX3	ENSG00000163661	727.2	953.4	1.3	*nd*	*nd*	*nd*
RELA	ENSG00000173039	75.5	94.0	1.2	*nd*	*nd*	*nd*
STAT1	ENSG00000115415	228.8	258.7	1.1	*nd*	*nd*	*nd*
STAT3	ENSG00000168610	51.0	61.1	1.2	*nd*	*nd*	*nd*
STC1	ENSG00000159167	*nd*	*nd*	*nd*	9.2	17.9	1.9
STC1	ENSG00000159167	28.7	52.7	1.8	*nd*	*nd*	*nd*
TGFB1	ENSG00000105329	157.3	120.0	0.8	273.7	191.8	0.7
TNFAIP6	ENSG00000123610	64.1	148.6	2.3	*nd*	*nd*	*nd*
TNFRSF1A	ENSG00000067182	153.4	140.6	0.9	*nd*	*nd*	*nd*
TSLP	ENSG00000145777	0.3	2.3	6.9	4.8	11.4	2.4
ZC3H12A	ENSG00000163874	8.7	17.1	2.0	*nd*	*nd*	*nd*
ZFP36	ENSG00000128016	53.5	89.9	1.7	*nd*	*nd*	*nd*

Legend: TPM counts: transcripts per million; nd: not detected.

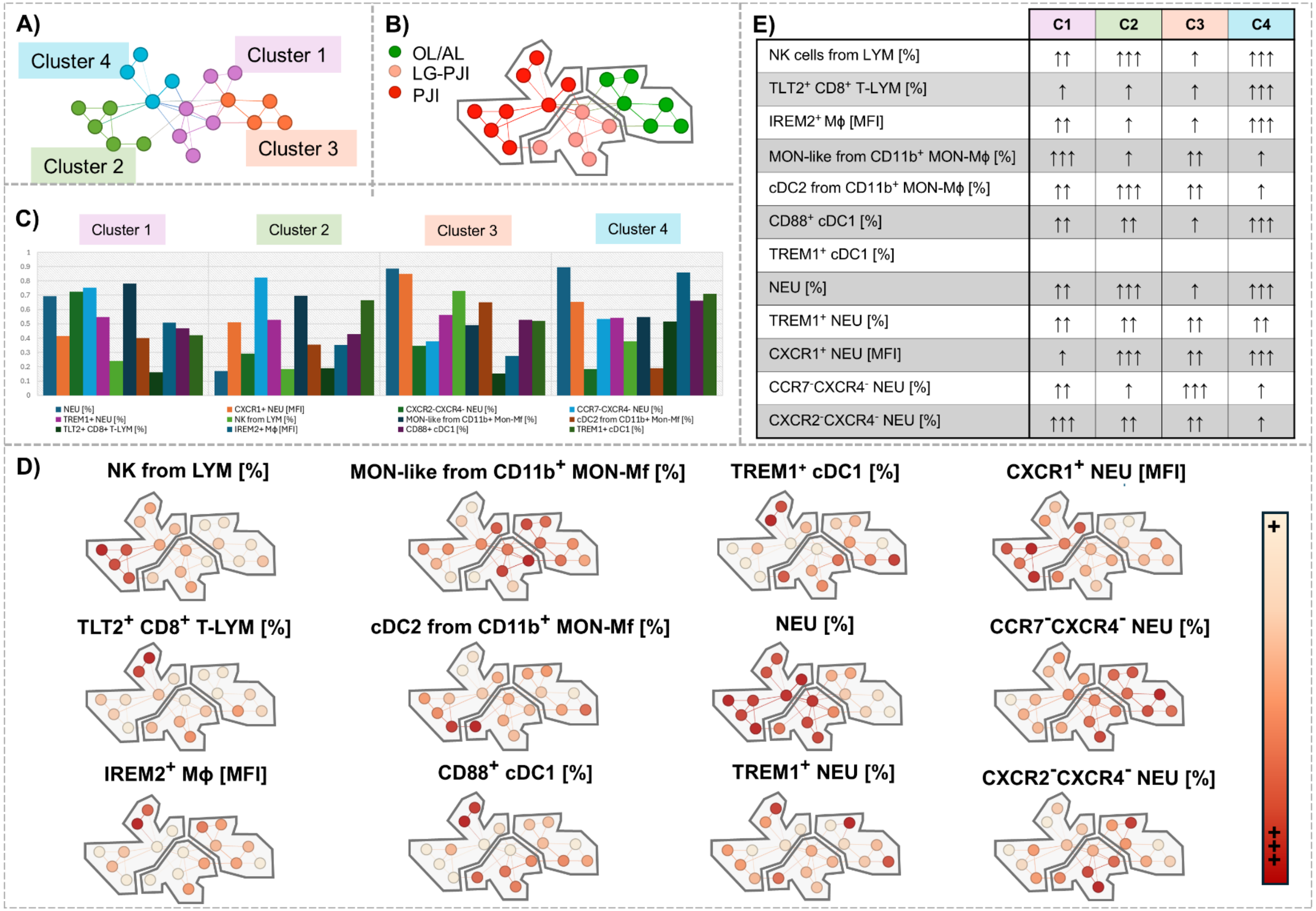

	C1	C2	C3	C4
NK cells from LYM [%]	↑↑	↑↑↑	↑	↑↑↑
TLT2+ CD8+ T-LYM [%]	↑	↑	↑	↑↑↑
IREM2+ Mϕ [MFI]	↑↑	↑	↑	↑↑↑
MON-like from CD11b+ MON-Mϕ [%]	↑↑↑	↑	↑↑	↑
cDC2 from CD11b+ MON-Mϕ [%]	↑↑	↑↑↑	↑↑	↑
CD88+ cDC1 [%]	↑↑	↑↑	↑	↑↑↑
TREM1+ cDC1 [%]				
NEU [%]	↑↑	↑↑↑	↑	↑↑↑
TREM1+ NEU [%]	↑↑	↑↑	↑↑	↑↑
CXCR1+ NEU [MFI]	↑	↑↑↑	↑↑	↑↑↑
CCR7-CXCR4- NEU [%]	↑↑	↑	↑↑↑	↑
CXCR2-CXCR4- NEU [%]	↑↑↑	↑↑	↑↑	↑

Supplementary Figure S5. Patient similarity network constructed based on the similarity of synovial fluid (SF)-derived immune cell parameters in patients with prosthetic joint infection (microbiologically confirmed; PJI), low-grade infection (LG-PJI) and aseptic conditions (osteolysis/ aseptic loosening; OL/AL). **(A)** Four clusters based on the similarity of SF-derived immune cell parameters were obtained. **(B)** Distribution of patient subgroups in the network. **(C)** Distribution of immune cell populations in particular clusters. Y axis shows the average values of the used markers/cell counts normalised to the maximum value in the data set. **(D)** Distribution of parameters used for network construction. Subgroups of patients with OL/AL, PJI and LG-PJI are marked by the grey outline. **(F)** Distribution of selected parameters between clusters. For numerical values of particular immune cell populations, 95% confidence intervals and P-values, see **Supplementary Table S6** and **Supplementary Table S7**.
Legend: ↑↑↑ highest, ↑↑ moderate, ↑ lowest percentage/MFI expression of markers or cell population within the data set.

Supplementary Figure S6. Distribution of dendritic cell subset types cDC1 and cDC2 (counted from monocyte–macrophage cell lineage) in groups of patients with PJI-all, OL/AL, OA and OA-INF. **Legend: OA:** patients with osteoarthritis; **OA-INF:** patients with osteoarthritis and infection; **OL/AL:** osteolysis/aseptic loosening; **LG-PJI:** low-grade prosthetic infection; **PJI:** microbiologically confirmed prosthetic infection.

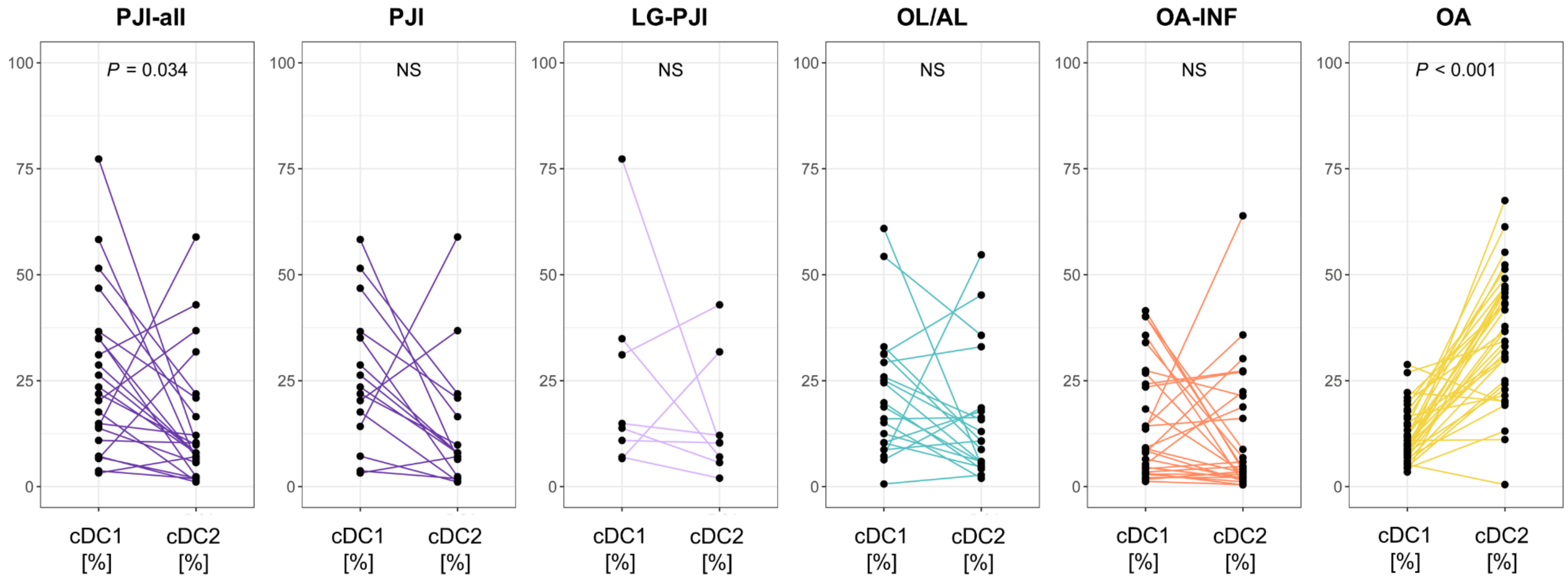

Supplementary Table S8. Distribution of synovial fluid (SF)-derived immune cell parameters and concentration of soluble mediators in SF between clusters of patients with prosthetic joint infection (microbiologically confirmed; PJI), low-grade infection (LG-PJI) and aseptic conditions (osteolysis/ aseptic loosening; OL/AL). Marked parameters have been used for network construction. The characteristics of clusters is shown in **Supplementary Figure S3.**

	**C1**	**C2**	**C3**	**C4**
No. of patients	6	5	4	4
Sex (men/women)	6/0	2/3	3/1	4
Knee/ hip	4/2	5/1	3/1	4/0
Age at SF sampling (years):<br>**mean** ±95% CI; [min–max]	**66.3** ± 13.1;<br>[44.0–78.0]; 0	**65.2** ± 11.7;<br>[50.0–74.0]; 0	**72.5** ± 15.7;<br>[63.0–86.0]; 0	**75.8** ± 8.5;<br>[69.0–80.0]; *0*
Time since joint difficulties started in months:<br>**mean** ±95% CI; [min–max]; *NA*	**24.3** ± 27.2;<br>[2.0–72.0]; 0	**6.5** ± 6.9;<br>[1.0–10.0]; 1	**39.0** ± 28.6;<br>[24.0–60.0]; 0	**10.2** ± 16.4;<br>[1.0–24.0]; *0*
Time since implant placement in months:<br>**mean** ±95% CI; [min–max]; *NA*	**9.8** ± 8.0;<br>[4.0–24.0]; 0	**12.0** ± 0.0;<br>[12.0–12.0]; 1	**3.8** ± 3.5;<br>[1.0–6.0]; 0	**25.2** ± 38.9;<br>[5.0–60.0]; *0*
Cement YES/NO/HYBRID /*NA*	4/1/1/*0*	3/1/0/*1*	3/1/0/*0*	4/0/0/*0*
Cement type: Biomet Optipac/ Copal G+C/ Refobacin /none/*NA*	2/1/2/*1*	0/0/1/1/*3*	2/0/1/1/*0*	1/0/1/0/*2*
BMI [kg/m^2]:<br>**mean** ±95% CI; [min–max]; *NA*	**31.2** ± 6.0;<br>[26.0–39.5]; 0	**31.7** ± 10.8;<br>[22.0–45.9]; 0	**28.3** ± 4.7;<br>[24.1–31.0]; 0	**28.1** ± 4.5;<br>[25.5–31.6]; *0*
Pain level in VAS[a]:<br>**mean** ±95% CI; [min–max]	**5.0** ± 1.3;<br>[4.0–7.0]; 0	**4.6** ± 1.4;<br>[3.0–6.0]; 0	**4.2** ± 2.4;<br>[3.0–6.0]; 0	**3.8** ± 1.5;<br>[3.0–5.0]; *0*
Pain level[b]: 0/1/2/3/*NA*	0/0/6/0/*0*	0/0/3/2/*0*	0/1/1/2/*0*	0/0/3/1/*0*
Fluid volume [ml]:<br>**mean** ±95% CI; [min–max]	**24.1** ± 25.9;<br>[3.0–65.0]; 0	**13.2** ± 12.7;<br>[2.0–30.0]; 0	**17.2** ± 19.2;<br>[6.0–30.0]; 0	**14.8** ± 14.8;<br>[5.0–25.0]; *0*
Viscosity: low/normal/high	6/0/0	2/3/0	2/2/0	1/2/1
Colour: transparent/yellow/cloudy/orange/*NA*	0/0/6/0/*0*	0/0/4/1/*0*	0/0/4/0/*0*	0/0/3/1/*0*
Absolute counts of white cells in SF x10^9/L:<br>**mean** ±95% CI; [min–max]	**3.7** ± 3.7;<br>[0.5–9.7]; *0*	**47.5** ± 60.9;<br>[0.6–118.0]; *0*	**1.0** ± 0.9;<br>[0.5–1.6]; *0*	**27.7** ± 28.1;<br>[13.5–52.8]; *0*
Percentage of CD45$^+$ cells [%]:<br>**mean** ± 95% CI; [min–max]	**18.2** ± 11.7;<br>[2.5–30.3]; *0*	**31.9** ± 34.4;<br>[5.3–75.8]; *0*	**26.1** ± 60.5;<br>[4.6–83.1]; *0*	**62.5** ± 22.3;<br>[41.6–70.6]; *0*
LYM [%]:<br>**mean** ±95% CI; [min–max]	**14.4** ± 12.1;<br>[3.1–28.5]; *0*	**5.2** ± 7.2;<br>[0.5–13.4]; *0*	**48.5** ± 52.0;<br>[6.4–86.1]; *0*	**2.6** ± 2.0;<br>[0.8–3.9]; *0*
MON-Mϕ [%]:<br>**mean** ±95% CI; [min–max]	**16.2** ± 12.3;<br>[1.0–29.4]; *0*	**6.9** ± 10.0;<br>[0.5–18.9]; *0*	**33.6** ± 49.4;<br>[6.0–77.9]; *0*	**8.7** ± 13.0;<br>[1.2–17.1]; *0*
NEU [%]:<br>**mean** ±95% CI; [min–max]	**68.1** ± 23.3;<br>[41.6–92.2]; *0*	**86.9** ± 16.1;<br>[65.9–98.1]; *0*	**16.5** ± 12.2;<br>[7.3–24.8]; *0*	**87.8** ± 14.9;<br>[79.6–97.9]; *0*
EOS-like cells [%]:<br>**mean** ± 95% CI; [min–max]	**1.6** ± 2.3;<br>[0.3–6.0]; *0*	**7.9** ± 10.7;<br>[1.9–14.3]; *1*	**0.5** ± 1.0;<br>[0.0–1.4]; *0*	**3.7** ± 3.3;<br>[1.5–6.1]; *0*
CD4$^+$/CD8$^+$ T-LYM [ratio]:<br>**mean** ± 95% CI; [min–max]	**1.8** ± 0.6;<br>[1.1–2.5]; *0*	**4.9** ± 4.4;<br>[2.5–8.8]; *1*	**2.4** ± 3.9;<br>[0.6–5.9]; *0*	**2.3** ± 0.9;<br>[1.7–3.0]; *0*
TLT-2$^+$ CD45RO$^+$ CD4$^+$ T-LYM [%]:<br>**mean** ± 95% CI; [min–max]; *NA*	**2.6** ± 2.6;<br>[0.2–6.0]; *0*	**2.5** ± 2.2;<br>[0.0–4.5]; *0*	**2.4** ± 4.9;<br>[0.5–4.5]; *1*	**8.8** ± 8.6;<br>[2.4–15.1]; *0*
TLT-2$^+$ CD8$^+$ T-LYM [%]:<br>**mean** ± 95% CI; [min–max]; *NA*	**4.9** ± 5.7;<br>[0.6–13.7]; *0*	**3.8** ± 3.5;<br>[0.0–7.2]; *0*	**5.9** ± 12.3;<br>[3.0–11.6]; *1*	**15.9** ± 22.9;<br>[1.4–30.8]; *0*
NK cells from LYM:<br>**mean** ± 95% CI; [min–max]	**10.0** ± 7.8;<br>[2.5–19.1]; *0*	**30.2** ± 15.9;<br>[8.0–41.4]; *0*	**7.6** ± 6.1;<br>[4.4–12.0]; *0*	**15.5** ± 5.1;<br>[12.6–18.6]; *0*
HLA-DR$^+$ NK [%]:<br>**mean** ± 95% CI; [min–max]	**15.2** ± 5.7;<br>[5.9–20.5]; *0*	**12.1** ± 21.3;<br>[0.6–31.3]; *1*	**23.1** ± 12.5;<br>[14.1–32.6]; *0*	**14.7** ± 14.2;<br>[6.8–27.5]; *0*
Mϕ [%]:<br>**mean** ± 95% CI; [min–max]	**1.2** ± 1.1;<br>[0.1–3.2]; *0*	**0.6** ± 0.9;<br>[0.0–1.7]; *0*	**12.4** ± 29.3;<br>[0.2–39.7]; *0*	**5.5** ± 9.8;<br>[0.1–12.0]; *0*

MON-like cells [%]:<br>**mean** ± 95% CI; [min–max]	**9.2** ± 7.9;<br>[0.5–17.4]; *0*	**1.8** ± 2.2;<br>[0.1–3.9]; *0*	**15.8** ± 21.8;<br>[2.3–34.7]; *0*	**2.2** ± 2.9;<br>[0.7–4.6]; *0*
cDC2 [%]:<br>**mean** ± 95% CI; [min–max]	**5.5** ± 4.7;<br>[0.3–10.7]; *0*	**4.4** ± 8.0;<br>[0.2–15.3]; *0*	**5.2** ± 5.8;<br>[2.8–10.6]; *0*	**0.6** ± 0.5;<br>[0.4–1.0]; *0*
HLA-DR$^+$ CD11b$^+$ Mon-Mϕ [MFI]:<br>**mean** ±95% CI; [min–max]	**29,393** ± 12,875;<br>[17,447–51,608]; *0*	**20,491** ± 13,953;<br>[9,997–30,323]; *1*	**34,427** ± 47,355;<br>[9,677–74,171]; *0*	**26,979** ± 28,479;<br>[7,927–47,684]; *0*
Mϕ from CD11b$^+$ Mon-Mϕ [%]:<br>**mean** ± 95% CI; [min–max]	**11.7** ± 14.8;<br>[3.4–39.8]; *0*	**10.9** ± 14.7;<br>[1.3–29.6]; *0*	**23.6** ± 32.9;<br>[3.5–51.0]; *0*	**42.1** ± 49.5;<br>[9.3–69.9]; *0*
MON-like cells from CD11b$^+$ Mon-Mϕ [%]:<br>**mean** ± 95% CI; [min–max]	**52.4** ± 15.3;<br>[25.6–67.1]; *0*	**33.0** ± 16.7;<br>[17.9–47.1]; *0*	**46.7** ± 9.8;<br>[39.0–52.8]; *0*	**36.8** ± 26.8;<br>[18.9–55.7]; *0*
cDC2 from CD11b$^+$ Mon-Mϕ [%]:<br>**mean** ± 95% CI; [min–max]	**32.4** ± 7.0;<br>[24.7–41.9]; *0*	**52.6** ± 29.7;<br>[22.7–80.8]; *0*	**28.6** ± 36.5;<br>[3.6–56.5]; *0*	**15.3** ± 20.9;<br>[2.8–32.2]; *0*
CD88$^+$ MON-like cells [%]:<br>**mean** ± 95% CI; [min–max]; *NA*	**81.1** ± 19.3;<br>[62.9–98.2]; *1*	**73.7** ± 135.3;<br>[63.0–84.3]; *3*	**70.0** ± 64.9;<br>[45.7–97.6]; *1*	**99.6** ± nan;<br>[99.6–99.6]; *3*
TLT-2$^+$ Mϕ [%]:<br>**mean** ± 95% CI; [min–max]; *NA*	**5.2** ± 9.0;<br>[0.5–13.4]; *2*	3.0 ± 8.4;<br>[2.3–3.6]; *3*	**3.9** ± 10.8;<br>[0.3–8.7]; *1*	**32.6** ± 24.1;<br>[30.7–34.5]; *2*
IREM-2$^+$ Mϕ [MFI]:<br>**mean** ± 95% CI; [min–max]; *NA*	**10,243** ± 2,623;<br>[8,993–12,640]; *2*	**5,568** ± 9,746;<br>[4,801–6,335]; *3*	**7,098** ± 3,695;<br>[6,173–8,814]; *1*	**17,317** ± 36,435;<br>[14,449–20,184]; *2*
CD88$^+$ cDC1 [%]:<br>**mean** ± 95% CI; [min–max]	**43.1** ± 22.8;<br>[22.4–60.3]; *1*	**48.4** ± 232.5;<br>[30.1–66.7]; *3*	**39.3** ± 32.2;<br>[26.4–52.3]; *1*	**60.8** ± 113.7;<br>[8.3–91.9]; *1*
TREM1$^+$ cDC1 [%]:<br>**mean** ± 95% CI; [min–max]	**38.8** ± 23.0;<br>[15.2–62.4]; *1*	**48.0** ± 296.1;<br>[24.7–71.3]; *3*	**61.1** ± 48.8;<br>[43.3–82.2]; *1*	**65.3** ± 83.8;<br>[27.5–92.2]; *1*
CD11b$^+$ NEUs [MFI]:<br>**mean** ± 95% CI; [min–max]; *NA*	**8,074** ± 1,632;<br>[5,984–10,378]; *0*	**11,105** ± 4,322;<br>[8,440–14,559]; *1*	**6,317** ± 2,176;<br>[4,966–7,621]; *0*	**8,631** ± 5,136;<br>[5,075–12,913]; *0*
CD88$^+$ NEUs [%]:<br>**mean** ± 95% CI; [min–max]	**49.9** ± 33.7;<br>[7.3–90.7]; *0*	**47.3** ± 20.5;<br>[27.9–68.1]; *0*	**58.1** ± 63.6;<br>[14.3–92.1]; *0*	**57.2** ± 40.1;<br>[24.1–85.2]; *0*
TREM1$^+$ NEUs [%]:<br>**mean** ± 95% CI; [min–max]	**51.2** ± 23.4;<br>[27.1–93.3]; *0*	**52.5** ± 21.4;<br>[25.8–71.6]; *0*	**49.4** ± 32.9;<br>[32.4–79.3]; *0*	**50.5** ± 43.2;<br>[28.2–85.7]; *0*
TLT-2$^+$ NEUs [%]:<br>**mean** ± 95% CI; [min–max]	**2.5** ± 2.5;<br>[0.2–5.2]; *0*	**7.9** ± 7.1;<br>[2.8–16.6]; *0*	**1.0** ± 1.0;<br>[0.2–1.7]; *0*	**4.1** ± 7.6;<br>[0.7–11.2]; *0*
CCR1$^+$ NEUs [%]:<br>**mean** ± 95% CI; [min–max]	**47.9** ± 9.5;<br>[33.1–57.0]; *0*	**70.4** ± 5.6;<br>[63.9–76.4]; *0*	**46.9** ± 16.0;<br>[38.0–61.1]; *0*	**52.7** ± 19.5;<br>[44.5–70.5]; *0*
CCR2$^+$ NEUs [%]:<br>**mean** ± 95% CI; [min–max]	**15.5** ± 12.3;<br>[0.3–33.0]; *0*	**52.8** ± 15.9;<br>[34.8–64.9]; *0*	**10.2** ± 8.2;<br>[5.1–17.3]; *0*	**31.1** ± 19.6;<br>[16.2–46.1]; *0*
CCR5$^+$ NEUs [%]:<br>**mean** ± 95% CI; [min–max]	**62.2** ± 18.9;<br>[32.5–81.8]; *0*	**64.6** ± 14.7;<br>[47.8–78.9]; *0*	**73.5** ± 29.0;<br>[48.8–91.5]; *0*	**64.8** ± 39.4;<br>[34.9–85.3]; *0*
CCR7$^+$ NEUs [%]:<br>**mean** ± 95% CI; [min–max]	**32.1** ± 8.1;<br>[23.1–42.2]; *0*	**49.9** ± 12.6;<br>[32.6–59.1]; *0*	**23.1** ± 9.6;<br>[17.6–29.3]; *0*	**38.6** ± 10.9;<br>[31.7–47.4]; *0*
CXCR1$^+$ NEUs [%]:<br>**mean** ± 95% CI; [min–max]	**75.8** ± 13.7;<br>[55.7–91.7]; *0*	**95.1** ± 6.0;<br>[88.7–99.9]; *0*	**95.5** ± 6.4;<br>[91.2–99.0]; *0*	**94.4** ± 5.7;<br>[90.1–98.1]; *0*
CXCR1$^+$ NEU [MFI]:<br>**mean** ± 95% CI; [min–max]	**5,744** ± 1,673;<br>[3,497–7,430]; *0*	**11,816** ± 2,282;<br>[9,620–13,892]; *0*	**7084** ± 2,344;<br>[5,592–9,115]; *0*	**9,058** ± 3,596;<br>[6,160–11,534]; *0*
CXCR2$^+$ NEUs [%]:<br>**mean** ± 95% CI; [min–max]	**59.1** ± 9.2;<br>[50.9–75.9]; *0*	**76.5** ± 6.6;<br>[70.1–82.8]; *0*	**75.6** ± 25.9;<br>[55.8–95.7]; *0*	**85.8** ± 10.6;<br>[80.8–95.0]; *0*
CXCR2$^+$ NEUs [MFI]:<br>**mean** ± 95% CI; [min–max]	**2,115** ± 260;<br>[1,839–2,410]; *0*	**3,075** ± 958;<br>[2,346–4,099]; *0*	**2,089** ± 672;<br>[1,731–2,583]; *0*	**3,302** ± 888;<br>[2,788–4,057]; *0*
CXCR4$^+$ NEUs [%]:<br>**mean** ± 95% CI; [min–max]	**53.8** ± 8.7;<br>[42.9–61.0]; *0*	**81.1** ± 12.6;<br>[73.3–97.9]; *0*	**53.9** ± 34.7;<br>[25.6–76.6]; *0*	**74.0** ± 17.5;<br>[62.9–89.1]; *0*
CXCR1$^-$ CCR2$^+$ NEUs [%]:<br>**mean** ± 95% CI; [min–max]	**0.0** ± 0.0;<br>[0.0–0.0]; *0*	**0.0** ± 0.0;<br>[0.0–0.0]; *0*	**0.0** ± 0.0;<br>[0.0–0.0]; *0*	**0.0** ± 0.0;<br>[0.0–0.0]; *0*
CXCR1$^+$ CCR2$^+$ NEUs [%]:<br>**mean** ± 95% CI; [min–max]	**15.2** ± 12.2;<br>[0.3–32.7]; *0*	**52.4** ± 16.3;<br>[34.0–64.7]; *0*	**9.9** ± 8.1;<br>[4.8–16.9]; *0*	**30.8** ± 19.6;<br>[15.9–45.7]; *0*

CXCR1$^+$ CCR2$^-$ NEUs [%]: **mean** ± 95% CI; [min–max]	**57.8** ± 12.8; [42.4–79.1]; *0*	**42.0** ± 19.5; [25.8–65.4]; *0*	**84.4** ± 14.6; [73.9–93.7]; *0*	**62.4** ± 25.5; [42.2–81.5]; *0*
CXCR1$^-$ CCR2$^-$ NEUs [%]: **mean** ± 95% CI; [min–max]	**27.0** ± 14.5; [10.2–48.2]; *0*	**5.6** ± 6.7; [0.2–12.4]; *0*	**5.7** ± 7.7; [1.5–10.6]; *0*	**6.8** ± 6.8; [2.6–12.1]; *0*
CXCR2$^-$ CXCR4$^+$ NEUs [%]: **mean** ± 95% CI; [min–max]	**3.6** ± 3.2; [0.1–7.7]; *0*	**4.9** ± 4.6; [2.1–10.8]; *0*	**3.2** ± 6.1; [0.0–8.2]; *0*	**2.6** ± 3.0; [0.5–4.9]; *0*
CXCR2$^+$ CXCR4$^+$ NEUs [%]: **mean** ± 95% CI; [min–max]	**46.6** ± 7.0; [38.5–55.2]; *0*	**74.3** ± 8.9; [68.8–86.4]; *0*	**45.9** ± 30.9; [20.1–66.9]; *0*	**68.7** ± 18.5; [57.5–84.5]; *0*
CXCR2$^+$ CXCR4$^-$ NEUs [%]: **mean** ± 95% CI; [min–max]	**21.8** ± 5.9; [14.6–28.9]; *0*	**7.4** ± 6.8; [1.9–16.2]; *0*	**39.6** ± 42.8; [20.4–78.1]; *0*	**21.5** ± 10.9; [12.4–28.9]; *0*
CXCR2$^-$ CXCR4$^-$ NEUs [%]: **mean** ± 95% CI; [min–max]	**28.1** ± 9.8; [14.5–38.7]; *0*	**13.4** ± 12.2; [0.9–22.7]; *0*	**11.3** ± 13.4; [1.8–20.3]; *0*	**7.1** ± 6.8; [0.9–10.5]; *0*
CCR7$^-$ CXCR4$^+$ NEUs [%]: **mean** ± 95% CI; [min–max]	**5.4** ± 4.9; [0.5–13.8]; *0*	**21.2** ± 22.5; [2.7–41.6]; *0*	**8.8** ± 16.2; [0.1–22.9]; *0*	**18.1** ± 18.2; [5.3–31.6]; *0*
CCR7$^+$ CXCR4$^+$ NEUs [%]: **mean** ± 95% CI; [min–max]	**21.1** ± 7.2; [11.2–29.8]; *0*	**42.0** ± 15.1; [23.1–51.4]; *0*	**14.1** ± 14.5; [2.5–24.2]; *0*	**32.0** ± 4.8; [28.8–35.4]; *0*
CCR7$^+$ CXCR4$^-$ NEUs [%]: **mean** ± 95% CI; [min–max]	**8.9** ± 6.5; [4.0–17.8]; *0*	**4.3** ± 7.2; [0.2–13.6]; *0*	**6.1** ± 7.1; [1.6–11.4]; *0*	**4.0** ± 4.7; [1.4–8.3]; *0*
CCR7$^-$ CXCR4$^-$ NEU [%]: **mean** ± 95% CI; [min–max]	**64.6** ± 7.6; [56.8–75.6]; *0*	**32.5** ± 14.7; [11.5–40.2]; *0*	**70.9** ± 17.3; [62.8–86.0]; *0*	**45.9** ± 15.5; [31.8–53.5]; *0*
CRP [µg/ml]: **mean** ± 95% CI; [min–max]	**7.3** ± 15.5; [0.0–37.0]; *0*	**36.9** ± 36.4; [0.0–81.6]; *0*	**4.8** ± 12.1; [0.0–16.1]; *0*	**15.4** ± 25.2; [0.0–36.4]; *0*
α-defensins [µg/ml]: **mean** ± 95% CI; [min–max]	**2.1** ± 3.5; [0.1–8.8]; *0*	**8.4** ± 7.2; [1.7–14.7]; *0*	**0.0** ± 0.0; [0.0–0.0]; *0*	**10.1** ± 6.6; [5.0–13.9]; *0*
PTX3 [ng/ml]: **mean** ± 95% CI; [min–max]	**5.6** ± 10.3; [0.0–25.0]; *0*	**82.9** ± 90.8; [7.5–162.2]; *0*	**8.7** ± 27.8; [0.0–34.9]; *0*	**67.3** ± 102.6; [0.0–129.7]; *0*
CXCL13 [pg/ml]: **mean** ± 95% CI; [min–max]	**1.4** ± 1.4; [0.2–3.6]; *0*	**2.5** ± 1.7; [0.2–3.7]; *0*	**0.1** ± 0.3; [0.0–0.3]; *0*	**2.2** ± 2.0; [1.0–3.8]; *0*
sCD14 [ng/ml]: **mean** ± 95% CI; [min–max]	**1.5** ± 0.1; [1.5–1.6]; *0*	**2.0** ± 0.5; [1.5–2.5]; *0*	**1.6** ± 0.5; [1.5–2.1]; *0*	**1.5** ± 0.0; [1.5–1.5]; *0*
sTREM1 [ng/ml]: **mean** ± 95% CI; [min–max]	**26.1** ± 4.0; [18.9–29.0]; *0*	**27.3** ± 17.0; [3.6–37.4]; *0*	**16.7** ± 7.9; [9.7–20.9]; *0*	**27.4** ± 9.3; [19.0–31.6]; *0*
PCT [pg/ml]: **mean** ± 95% CI; [min–max]	**276.4** ± 429.1; [0.0–1054.5]; *0*	**100.5** ± 223.4; [0.0–415.1]; *0*	**0.0** ± 0.0; [0.0–0.0]; *0*	**0.0** ± 0.0; [0.0–0.0]; *0*

Legend: T-LYM: T-lymphocytes, MON–Mϕ: monocyte–macrophage lineage; NEUs: neutrophils; Mϕ: macrophage; MON-like: monocyte-like cells; cDC1: conventional dendritic cells type 1; cDC2: conventional dendritic cells type 2; CD8$^+$ T-LYM: cytotoxic T-lymphocytes; CD4$^+$ T-LYM: helper T-lymphocytes.

Supplementary Table S9. Differences in distribution of demographic, laboratory and clinical parameters between clusters, obtained by a patient similarity network, of patients with prosthetic joint infection (microbiologically confirmed; PJI), low-grade infection (LG-PJI) and aseptic condition (osteolysis/ aseptic loosening; OL/AL). The characteristics of clusters are shown in **Supplementary Figure S8**. Significant P-values are marked in bold.

	Mann-Whitney U-test P-value					
	**C1 vs C2**	**C1 vs C3**	**C1 vs C4**	**C2 vs C3**	**C2 vs C4**	**C3 vs C4**
No. of patients	6 vs 5	6 vs 4	6 vs 4	5 vs 4	5 vs 4	4 vs 4
Sex (men/women)	6/0 vs 2/3	6/0 vs 3/1	6/0 vs 4/0	2/3 vs 3/1	2/3 vs 4/0	3/1 vs 4/0
Knee/ hip	4/2 vs 5/1	4/2 vs 3/1	4/2 vs 4/0	5/1 vs 3/1	5/1 vs 4/0	3/1 vs 4/0
Time since joint difficulties started [months]	0.209	0.133	0.237	**0.021**	0.620	0.081
Time since implant placement [months]	0.209	0.133	0.237	**0.021**	0.620	0.081
BMI [kg/m^2]	1.000	0.610	0.476	0.413	0.556	1.000
Total CD45$^+$ count [×10^9/l]	0.082	0.171	**0.010**	0.063	0.730	**0.029**
LYM [%]	0.126	0.067	**0.019**	0.063	1.000	**0.029**
MON-Mϕ [%]	0.170	0.610	0.476	0.065	0.712	0.114
NEU [%]	0.082	**0.010**	0.257	**0.016**	0.905	**0.029**
Percentage of CD45$^+$ cells [%]	0.329	0.762	**0.010**	0.556	0.190	0.343
EOS-like cells [%]	**0.038**	0.352	0.067	**0.029**	0.686	**0.029**
CD4$^+$/CD8$^+$ T-LYM [ratio]	**0.019**	0.762	0.257	0.200	0.114	0.686
TLT-2$^+$ CD45RO$^+$ CD4$^+$ T-LYM [%]	1.000	0.905	0.067	1.000	0.111	0.114
TLT-2$^+$ CD8$^+$ T-LYM [%]	0.792	0.714	0.171	0.786	0.286	0.629
NK cells from LYM [%]	**0.030**	1.000	0.476	0.063	0.190	**0.029**
HLA-DR$^+$ NK cells [%]	0.476	0.199	0.762	0.200	0.486	0.200
Mϕ [%]	0.247	0.171	0.762	0.111	0.286	0.886
MON-like cells [%]	0.082	0.610	0.257	0.063	0.730	0.114
cDC2 [%]	0.537	0.914	0.114	0.556	0.905	**0.029**
HLA-DR$^+$ CD11b$^+$ Mon-Mϕ [MFI]	0.352	0.914	0.762	0.886	0.886	0.886
Mϕ from CD11b$^+$ Mon-Mϕ [%]	0.931	0.352	0.067	0.413	0.111	0.486
Mon-like cells from CD11b$^+$ Mon-Mϕ [%]	**0.030**	0.352	0.171	0.190	0.730	0.686
cDC2 from CD11b$^+$ Mon-Mϕ [%]	0.177	0.762	**0.038**	0.190	**0.032**	0.486
CD88$^+$ MON-like cells [%]	0.857	0.571	0.333	1.000	0.667	0.500
TLT-2$^+$ Mϕ [%]	1.000	0.629	0.133	1.000	0.333	0.200
IREM-2$^+$ Mϕ [MFI]	0.133	0.057	0.133	0.800	0.333	0.200
CD88$^+$ cDC1 [%]	0.571	1.000	0.571	0.800	0.800	0.700
CD11b$^+$ NEUs [MFI]	0.067	0.171	0.762	**0.029**	0.200	0.200
CD88$^+$ NEUs [%]	0.931	0.610	0.762	0.806	0.730	0.886
TREM1$^+$ NEUs [%]	0.662	0.610	1.000	0.730	0.905	0.886
TLT-2$^+$ NEUs [%]	0.082	0.669	0.762	**0.016**	0.190	0.200
CCR1$^+$ NEUs [%]	**0.004**	0.762	0.914	**0.016**	0.085	0.486
CCR2$^+$ NEUs [%]	**0.004**	0.476	0.114	**0.016**	0.063	0.057
CCR5$^+$ NEUs [%]	1.000	0.476	0.610	0.286	0.905	0.886
CCR7$^+$ NEUs [%]	**0.030**	0.114	0.257	**0.016**	0.111	**0.029**
CXCR1$^+$ NEUs [%]	**0.009**	**0.019**	**0.019**	0.905	0.905	0.486
CXCR1$^+$ NEUs [MFI]	**0.004**	0.352	0.067	**0.016**	0.111	0.343
CXCR2$^+$ NEUs [%]	**0.030**	0.134	**0.010**	0.905	0.190	0.343
CXCR2$^+$ NEUs [MFI]	**0.009**	0.762	**0.010**	0.063	0.556	**0.029**
CXCR4$^+$ NEUs [%]	**0.004**	0.762	**0.010**	0.063	0.413	0.343
CXCR1$^-$ CCR2$^+$ NEUs [%]	0.191	0.331	0.271	0.893	1.000	0.869
CXCR1$^+$ CCR2$^+$ NEUs [%]	**0.004**	0.476	0.114	**0.016**	0.063	0.057
CXCR1$^+$ CCR2$^-$ NEUs [%]	0.126	**0.019**	0.476	**0.016**	0.190	0.114

CXCR1⁻ CCR2⁻ NEUs [%]	**0.009**	**0.019**	**0.019**	0.905	0.905	0.686
$CXCR2^-$ $CXCR4^+$ NEUs [%]	0.429	0.914	0.914	0.556	0.623	0.886
$CXCR2^+$ $CXCR4^+$ NEUs [%]	**0.004**	1.000	**0.010**	**0.016**	0.413	0.114
$CXCR2^+$ $CXCR4^-$ NEUs [%]	**0.009**	0.352	1.000	**0.016**	**0.032**	0.486
$CXCR2^-$ $CXCR4^-$ NEUs [%]	0.052	0.067	**0.010**	0.905	0.413	0.686
$CCR7^-$ $CXCR4^+$ NEUs [%]	0.126	0.762	0.114	0.286	1.000	0.343
$CCR7^+$ $CXCR4^+$ NEUs [%]	**0.030**	0.257	**0.019**	**0.032**	0.190	**0.029**
$CCR7^+$ $CXCR4^-$ NEUs [%]	0.247	0.476	0.114	0.413	0.556	0.686
$CCR7^-$ $CXCR4^-$ NEUs [%]	**0.004**	0.352	**0.010**	**0.016**	0.190	**0.029**
CRP [μg/ml]	0.135	0.660	0.379	0.140	0.325	0.384
α-defensins [μg/ml]	**0.030**	**0.010**	**0.038**	**0.016**	0.905	**0.029**
PTX3 [ng/ml]	**0.013**	0.570	0.235	**0.034**	0.556	0.219
CXCL13 [pg/ml]	0.247	**0.042**	0.352	**0.037**	1.000	**0.029**
sCD14 [ng/ml]	**0.030**	1.000	0.610	0.190	**0.032**	0.686
sTREM1 [ng/ml]	0.126	**0.038**	0.476	0.190	0.413	0.114
PCT [pg/ml]	0.690	0.149	0.149	0.240	0.240	1.000

Legend: T-LYM: T-lymphocytes, MON–Mϕ: monocyte–macrophage lineage; NEUs: neutrophils; Mϕ: macrophage; MON-like cells: monocyte-like cells; cDC1: conventional dendritic cells type 1; cDC2: conventional dendritic cells type 2; $CD8^+$ T-LYM: cytotoxic T-lymphocytes; $CD4^+$ T-LYM: helper T-lymphocytes; CRP: C-reactive protein; PTX3: pentatrexin 3; PCT: procalcitonin.

References:

1. McNally M, Sousa R, Wouthuyzen-Bakker M, et al. The EBJIS definition of periprosthetic joint infection. *The bone & joint journal* 2021;**103-B**(1):18-25.
